# Annexin-1 regulated by HAUSP is essential for UV-induced damage response

**DOI:** 10.1038/cddis.2015.32

**Published:** 2015-02-19

**Authors:** J-J Park, K-H Lim, K-H Baek

**Affiliations:** 1 Department of Biomedical Science, CHA University, Gyeonggi-Do 463-400, Republic of Korea

## Abstract

DNA damage can occur through diverse stimulations such as toxins, drugs, and environmental factors. To respond to DNA damage, mammalian cells induce DNA damage response (DDR). DDR signal activates a rapid signal transduction pathway, regulating the cell fate based on the damaged cell condition. Moreover, serious damaged cells have to be eliminated by the macrophage to maintain homeostasis. Because the DDR induces genomic instability followed by tumor formation, targeting the DDR signaling can be applied for the cancer therapy. Herpes virus-associated ubiquitin-specific protease (HAUSP/USP7) is one of the well-known deubiquitinating enzymes (DUBs) owing to its relevance with Mdm2-p53 complex. The involvement of HAUSP in DDR through p53 led us to investigate novel substrates for HAUSP, which is related to DDR or apoptosis. As a result, we identified annexin-1 (ANXA1) as one of the putative substrates for HAUSP. ANXA1 has numerous roles in cellular systems including anti-inflammation, damage response, and apoptosis. Several studies have demonstrated that ANXA1 can be modified in a post-translational manner by processes such as phosphorylation, SUMOylation, and ubiquitination. In addition, DNA damage gives various functions to ANXA1 such as stress response or cleavage-mediated apoptotic cell clearance. In the current study, our proteomic analysis using two-dimensional electrophoresis, matrix-assisted laser desorption/ionization-time-of-flight mass spectrometry (MALDI-TOF-MS) and nano LC-MS/MS, and immunoprecipitation revealed that ANXA1 binds to HAUSP through its HAUSP-binding motif (P/AXXS), and the cleavage and damage-responsive functions of ANXA1 upon UV-induced DNA damage may be followed by HAUSP-mediated deubiquitination of ANXA1. Intriguingly, the UV-induced damage responses via HAUSP-ANXA1 interaction in HeLa cells were different from the responses shown in the Jurkat cells, suggesting that their change of roles may depend on the cell types.

Most proteins follow the ubiquitin-proteasome pathway (UPP) to degradation; this involves successive enzymatic activities of the E1, E2, and E3 enzymes. In addition to proteasomal degradation, the proteins obtain or alter their functions through mono- or polyubiquitination.^1^ Thus, the ‘ubiquitin tag' is considered as an important feature for intracellular homeostasis. Deubiquitination is a reversible process against ubiquitination that detaches ubiquitin molecules from ubiquitinated proteins, and the process of deubiquitination is mediated by specific enzymes called deubiquitinating enzymes (DUBs). To date, almost ~100 DUBs have been identified, and they are involved in various cellular functions through their capability by which they deubiquitinate and thereby stabilize or alter the functions of their target proteins.^2^ DUBs are composed of at least six subfamilies: ubiquitin-specific proteases (USPs), ubiquitin C-terminal hydrolases (UCHs), ovarian tumor (OTU), Machado-Josephin domain papain-like cysteine proteases (MJDs), JAB1/MPN/Mov34 metalloenzyme (JAMM) domain zinc-dependent metalloprotease family, and monocyte chemotactic protein-induced proteases (MCPIPs).^3^ In addition, DUBs share specific regions including Cys, Asp/Asn, and His boxes for their deubiquitinating activities.^4^ The USP family has the most number among DUBs (~58 USPs),^5^ and many studies have demonstrated that human USPs have important roles in a broad range of cellular systems.^6^ In particular, their involvement in cell proliferation, signal transduction, and apoptosis emphasizes that abnormal or deregulated functions of USPs can be related to severe diseases including immune disorders and cancers.^2, 6, 7^ Accordingly, USPs have been widely targeted for the therapy of several diseases; however, a clear understanding of the molecular details underlining USPs and other DUBs has not yet been obtained.

HAUSP, also known as USP7, is a member of the USP family of DUBs. The importance of HAUSP in cells was demonstrated by its ability to specifically recognize and deubiquitinate both the tumor suppressor p53 and Mdm2, a p53-specific E3 ligase. In the normal state, HAUSP specifically binds to and deubiquitinates Mdm2, thereby stabilizing Mdm2 and subsequently inducing the proteasomal degradation of p53 through Mdm2 activity. Upon DNA damage, HAUSP is dephosphorylated by PPM1G. In this state, the deubiquitinating activity of HAUSP for Mdm2 decreases and HAUSP prefers p53 for its substrate instead of Mdm2. Such altered affinity of HAUSP to p53 leads to DNA repair and tumor-suppressive functions of p53.^8, 9, 10^ In addition to Mdm2 and p53, further studies have revealed that HAUSP can regulate various substrates, including ataxin-1, Chfr, claspin, Daxx, FOXO4, histone H2B, PTEN, NF-*κ*B, Tip60, UbE2E1, and UVSSA.^2^ These findings suggest that HAUSP has diverse roles in the cell through the regulation of different substrates and other additional proteins. In a present study, we performed two-dimensional gel electrophoresis (2-DE) and other proteomics-based experiments using HeLa cells to identify putative substrates regulated by HAUSP. We found several putative substrates, some of which are known to be involved in apoptosis or DNA damage response (DDR). Annexin a1, also known as ANXA1 and lipocortin 1, was also found as a putative binding partner for HAUSP, suggesting that ANXA1 may possibly be regulated by HAUSP-mediated deubiquitination.

Annexins consist of 13 annexin members and have four conserved repeated domains, which are responsible for Ca^2+^ and phospholipid binding. In most annexins, the conserved annexin domains enable them to bind the phospholipid of the membranes in a Ca^2+^-dependent manner, resulting in subsequent activities such as membrane trafficking, signal transduction, and exocytosis.^11^ However, major differences of annexins derive from their unique N-terminal regions. The N-terminus of each annexin member, which is responsible for specific functions, varies.^12^ ANXA1, the first member of the annexin superfamily, is a 37-kDa protein abundant in cells. Like other annexin proteins, ANXA1 binds to phospholipid in the presence of Ca^2+^.^13^ The biological functions of ANXA1 are extensively studied: anti-inflammatory mediator,^14, 15^ relationship with tumorigenesis,^16^ DDR,^17, 18^ and involvement in apoptosis and apoptotic cell clearance.^19, 20^ Another important feature of ANXA1 activity is the cleavage of the N-terminal region of ANXA1. When DNA damage or stress occurs, ANXA1 is cleaved by several proteases, resulting in the generation of the N-terminal fragment (Ac2-26) and cleaved form of ANXA1 (33 kDa). Importantly, both the full-length ANXA1 and Ac2-26 can be translocated to the cell membrane and induce apoptotic cell clearance by recruiting monocytes via chemoattraction.^20^ Thus, the ANXA1 cleavage process is considered essential for cell phagocytosis, as also revealed in neutrophil apoptosis and phagocytosis during inflammation.^14^ Otherwise, in response to cell damage, ANXA1 functions as a stress protein or a protective protein for DNA damage, resulting in nuclear localization of ANXA1.^18, 21, 22^ Overall, it is evident that ANXA1 participates in various cellular responses.

In the current study, we have identified ANXA1 as a novel substrate for HAUSP. HAUSP can bind to, deubiquitinate, and co-localize with ANXA1. Surprisingly, upon UV-induced DNA damage, the binding and the deubiquitinating activity of HAUSP to ANXA1 are increased. In addition, ANXA1 in HAUSP-deficient cells showed different localization and altered expression level and cleavage. Moreover, HAUSP-mediated regulation of ANXA1 shown in HeLa cells was different from the one in Jurkat cells. We found that apoptosis and transmigrative ratio of monocytes in HAUSP-depleted Jurkat cells coincides with the regulation of ANXA1 protein level and cleavage. Taken together, we suggest that ANXA1 functions of UV-induced DDR are regulated by the deubiquitinating activity of HAUSP.

## Results

### Identification of apoptosis-regulating proteins in HAUSP-overexpressing cells

**Figure 1:**
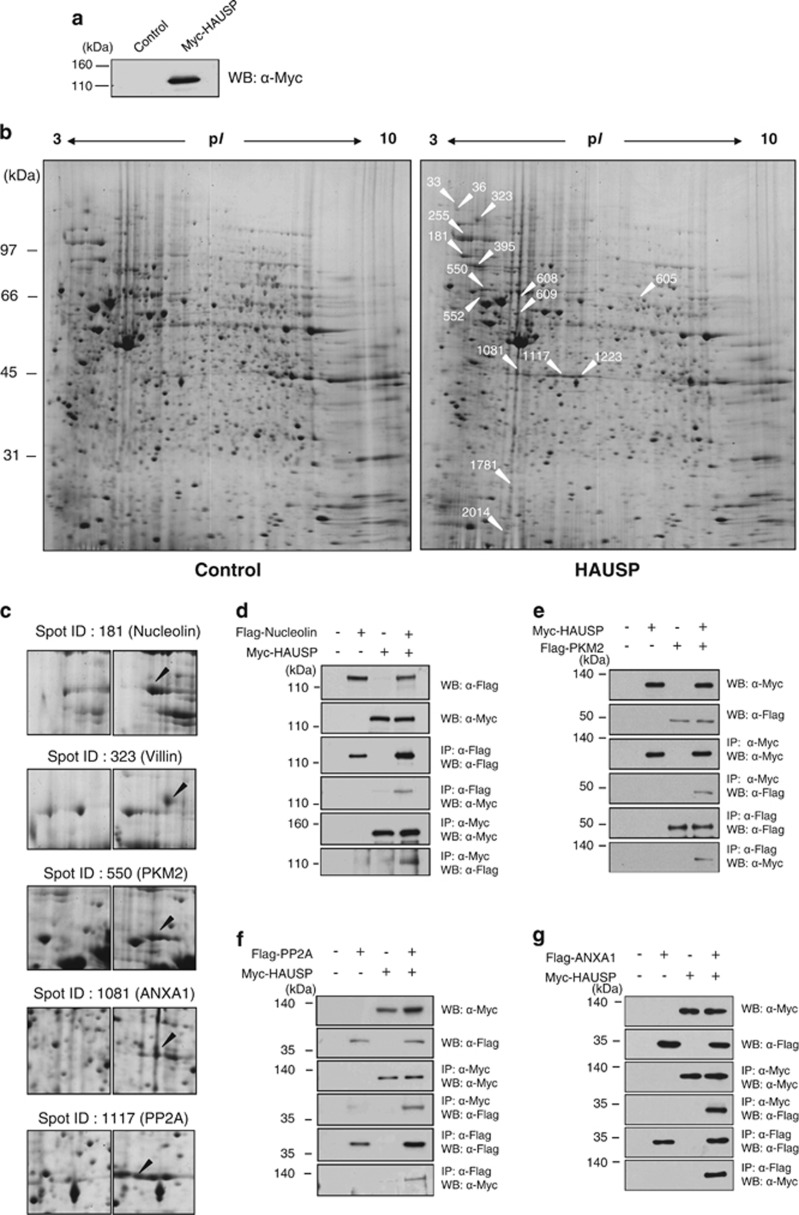
Identification of HAUSP interacting proteins. (**a**) HeLa cells expressing Myc-tagged HAUSP and these cell extracts were immunoblotted with an anti-Myc antibody. (**b**) Myc-tagged empty vector (left) and HAUSP (right) were transfected into HeLa cells. For the gel image analysis, we used a vector control (left) and HAUSP overexpression (right) lysates, and analyzed by 2-DE SDS-PAGE. The arrows indicated in the right panel represent all changed proteins as presented in Table 1. (**c**) Five apoptosis-related proteins are indicated. The arrows indicate in the right panel represent upregulated proteins by HAUSP overexpression compared with a control (left). (**d**–**g**) Myc-tagged HAUSP and Flag-tagged nucleolin, PKM2, PP2A, and ANXA1-overexpressing HEK 293T cell extracts were immunoprecipitated with an anti-Flag antibody. Then, the immunoprecipitation and protein expression were analyzed by immunoblotting with anti-Myc and anti-Flag antibodies. For the reciprocal immunoprecipitation, Myc-tagged HAUSP and Flag-tagged nucleolin, PKM2, PP2A, and ANXA1-overexpressing cell lysates were immunoprecipitated with an anti-Myc antibody followed by immunoblotting with an anti-Flag antibody

A previous study showed that the HAUSP overexpression regulates cancer cell proliferation and apoptosis through p53 signaling. ^9^ We also found previously that the overexpression of HAUSP leads to cancer cell apoptosis.^23, 24^ We explored the mechanisms involved in HAUSP-related cancer cell regulation by performing 2-DE-SDS-PAGE analysis using HAUSP-overexpressing cells (Figure 1a; Supplementary Figure S1). A total of 2095 protein spots were detected by Coomassie Brilliant Blue (CBB) G-250 (pH ranges 3–10) (Figure 1b). Comparative analysis between the control and HAUSP-overexpressing cells was conducted using a 2-DE gel image analysis program (Amersham, ImageMaster 2D Platinum, Piscataway, NJ, USA). We then determined a total of 16 upregulated proteins in the HAUSP-overexpressing cell lysate that showed 2- to 10-fold increases. Proteins were identified by tandem mass spectrometric analysis, and we assessed the identity of protein spots using the MASCOT search engine. The proteins identified based on probability-based scoring and spectra are summarized in Table 1 and Supplementary Figure S2. We used the high scores to select five proteins; nucleolin (spot ID 181), villin (spot ID 323), pyruvate kinase M2 (known as PKM2) (spot ID 550), ANXA1 (spot ID 1081), and PP2CA (known as PP2A) (spot ID 1117) (Figure 1c). We then checked the mRNA levels of these five proteins to evaluate the transcriptional regulation of the upregulated proteins (Supplementary Figure S3). Overexpression of HAUSP significantly increased villin mRNA level, but the other proteins (nucleolin, PKM2, ANXA1, and PP2A) did not show any significant change in their transcriptional levels (Supplementary Figure S3). The overexpression of HAUSP may, therefore, lead to the stabilization of nucleolin, PKM2, ANXA1, and PP2A by post-translational modification.

### ANXA1 interacts with HAUSP through its HAUSP-binding motif

**Figure 2:**
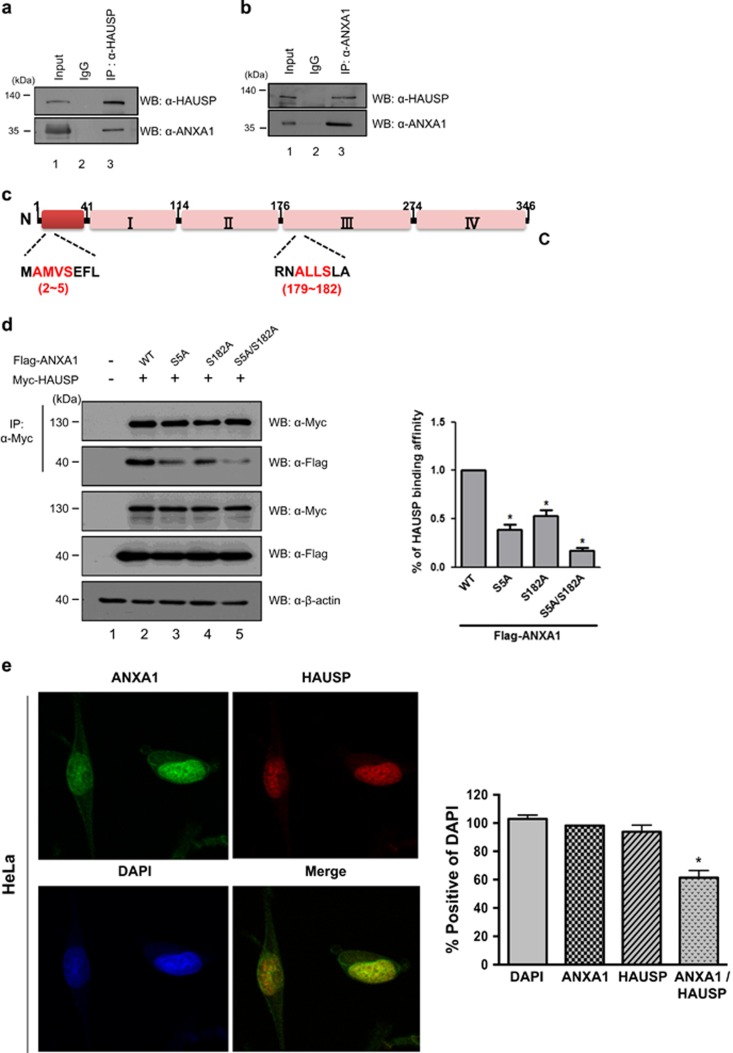
ANXA1 interacts with HAUSP via its HAUSP-binding motif. (**a**, **b**) HeLa cells were lysed, and proteins were immunoprecipitated with either an anti-HAUSP or an anti-ANXA1 antibody. Western blotting was performed using anti-HAUSP and anti-ANXA1 antibodies to detect endogenous binding between HAUSP and ANXA1. (**c**) Schematic of amino-acid structure of ANXA1. The amino-acid sequences showing HAUSP-binding motifs (AMVS and ALLS) are indicated in red color. (**d**) Myc-HAUSP and/or wild-type ANXA1, ANXA1 (S5A), ANXA1 (S182A), and ANXA1 (S5A/S182A) were transfected into HEK 293T cells. After lysis, cell lysates were immunoprecipitated with an anti-Myc antibody, and western blotting was performed using an anti-ANXA1 antibody to detect the binding affinity of ANXA1 to HAUSP. Statistical data are presented as a means (*n*=3, *P<0.05). (**e**) HeLa cells on coverglass were incubated with anti-HAUSP and anti-ANXA1 antibodies followed by FITC staining. DAPI was used for counterstaining of the nucleus (Green: ANXA1, Red: HAUSP, Blue: DAPI, Yellow: co-localization). The statistical data are representative of four biological replicates (*n*=4, *P<0.05)

We previously identified ANXA1 as one of candidates for HAUSP-binding partners (Figure 1g). Therefore, we next tested endogenous interaction between HAUSP and ANXA1. Their interaction was confirmed by endogenous immunoprecipitation (IP) in HeLa cells or *in vitro* biochemical assay with GST-tagged HAUSP, indicating that ANXA1 binds to HAUSP (Figures 2a and b; Supplementary Figure S4). Recently, an increasing line of evidence, namely, that substrates of HAUSP have amino-acid sequences for HAUSP-binding motifs (P/AXXS), has been reported.^8^ Because ANXA1 also has HAUSP-binding motif sequences (AMVS and ALLS) on its N-terminal region and annexin conserved domain (Figure 2c), we assumed that the binding of ANXA1 to HAUSP is mediated through HAUSP-binding motifs. Thus, we generated ANXA1 mutants that are mutated on its HAUSP-binding motif (S5A, S182A, and S5A/S182A) to investigate whether these motifs are important for interaction with HAUSP. ANXA1 mutants showed reduced binding affinity to HAUSP compared with wild-type ANXA1 (Figure 2d), demonstrating that HAUSP-binding motif on ANXA1 is indeed required to bind to HAUSP. Finally, the localization study by immunofluorescence analysis in HeLa cells showed that HAUSP and ANXA1 are co-localized in the nucleus (Figure 2e). Overall, our data clearly indicate that ANXA1 binds to HAUSP through its HAUSP-binding motifs and is co-localized with HAUSP in the nucleus.

### Deubiquitinating activity of HAUSP toward ANXA1

**Figure 3:**
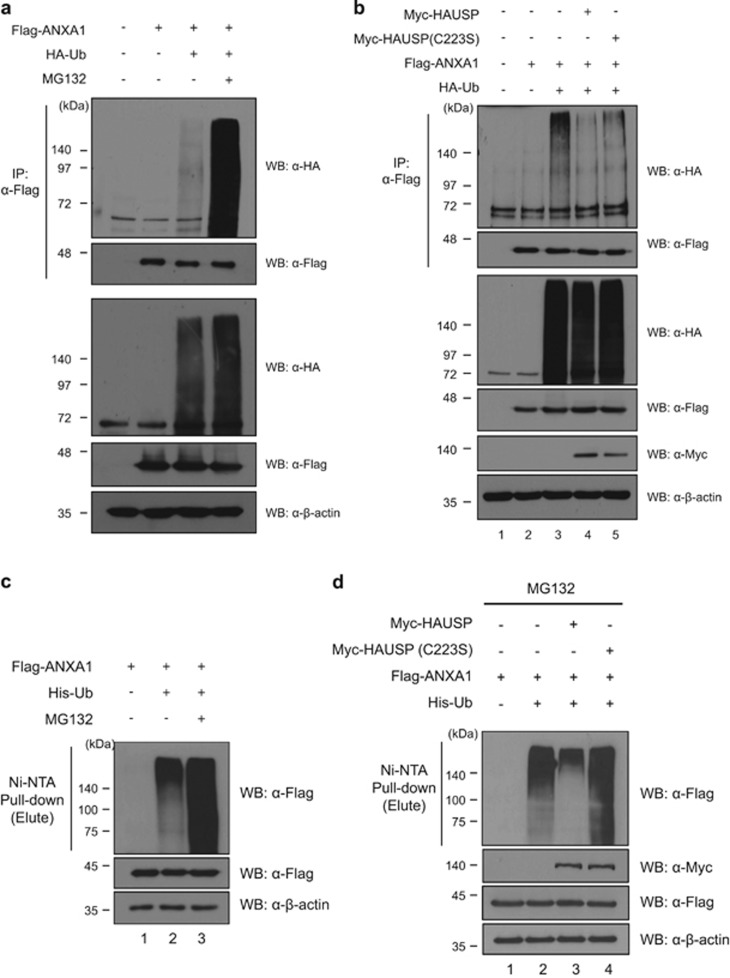
HAUSP deubiquitinates and stabilizes ANXA1. (**a**) The ubiquitination of ANXA1 was detected by immunoprecipitation with an anti-Flag antibody in Flag-ANXA1 and HA-ubiquitin transfected HEK 293T cell lysates. For inhibition of the 26S proteasome, 2.5 *μ*M of MG132, a proteasome inhibitor, was treated for 4 h before cell harvest. (**b**) HEK 293T cell lysates transfected with Myc-HAUSP or Myc-HAUSP catalytic mutant (C223S), Flag-ANXA1, and HA-ubiquitin were immunoprecipitated with an anti-Flag antibody. Subsequently, western blotting was performed to detect the ubiquitination level of ANXA1. (**c**) Cell lysates from HEK 293T cells transfected with Flag-ANXA1 and His-ubiquitin were subjected to ubiquitination assay with Ni-NTA beads. For inhibition of the 26S proteasome, 2.5 *μ*M of MG132 was treated for 4 h before cell harvest. Western blotting was performed with indicated antibodies. (**d**) Cell lysates from HEK 293T cells transfected with Myc-HAUSP or a catalytic mutant Myc-HAUSP (C223S), Flag-ANXA1, and His-ubiquitin were subjected to deubiquitination assay with Ni-NTA beads. The proteasome inhibitor MG132 was treated for 4 h before cell harvest. Western blotting was performed with indicated antibodies

It has been demonstrated that ANXA1 undergoes UPP;^25^ therefore, we hypothesize that HAUSP can protect ANXA1 from proteasomal degradation via the deubiquitinating capacity. We first reconfirmed the ubiquitination of ANXA1 (Figures 3a and c). The treatment of MG132, a proteasome inhibitor, dramatically increased ubiquitinated ANXA1. This suggests that ANXA1 may be degraded by the 26S proteasome. Next, we investigated the deubiquitinating capability of HAUSP toward ANXA1. Overexpression of HAUSP reduced the ubiquitination level of ANXA1 (Figures 3b and d). Moreover, dose-dependent overexpression of HAUSP increased the ANXA1 protein level, as confirmed by western blotting (Supplementary Figure S5). These results demonstrate that HAUSP is involved in the regulation for the ubiquitination of ANXA1.

### Deubiquitination of ANXA1 by HAUSP regulates UV-induced damage response in HeLa cells

**Figure 4:**
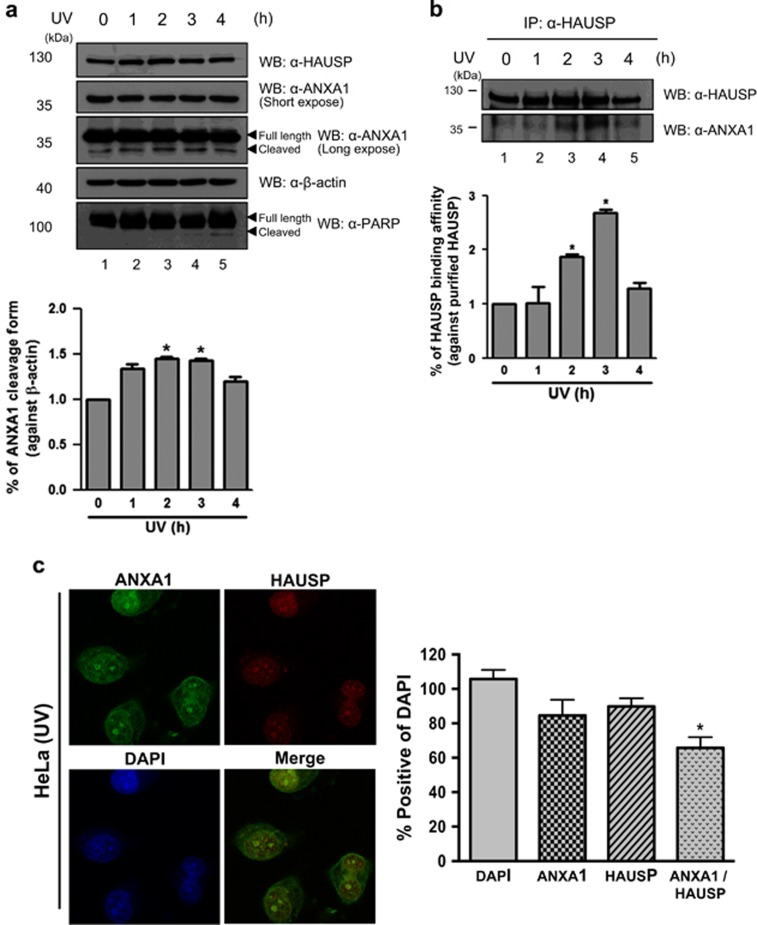
UV induces increased binding of HAUSP and ANXA1. (**a**) HeLa cells time-dependently treated with 30 mJ/cm^2^ of UV were lysed and ANXA1 protein levels were detected by western blotting. (**b**) HeLa cells described in (**a**) were lysed, and the interaction between HAUSP and ANXA1 was chased by immunoprecipitation using an anti-HAUSP antibody followed by western blotting using anti-HAUSP and anti-ANXA1 antibodies. PARP-1 antibody was used as a control for UV-induced damage. All statistical data are presented as a means (*n*=3, *P<0.05). (**c**) HeLa cells incubated for 3 h following UV were incubated with anti-HAUSP and anti-ANXA1 antibodies followed by FITC staining. DAPI was used for counterstaining of the nucleus (Green: ANXA1, Red: HAUSP, Blue: DAPI, and Yellow: co-localization). The data are representative of four biological replicates (*n*=4, *P<0.05)

**Figure 5:**
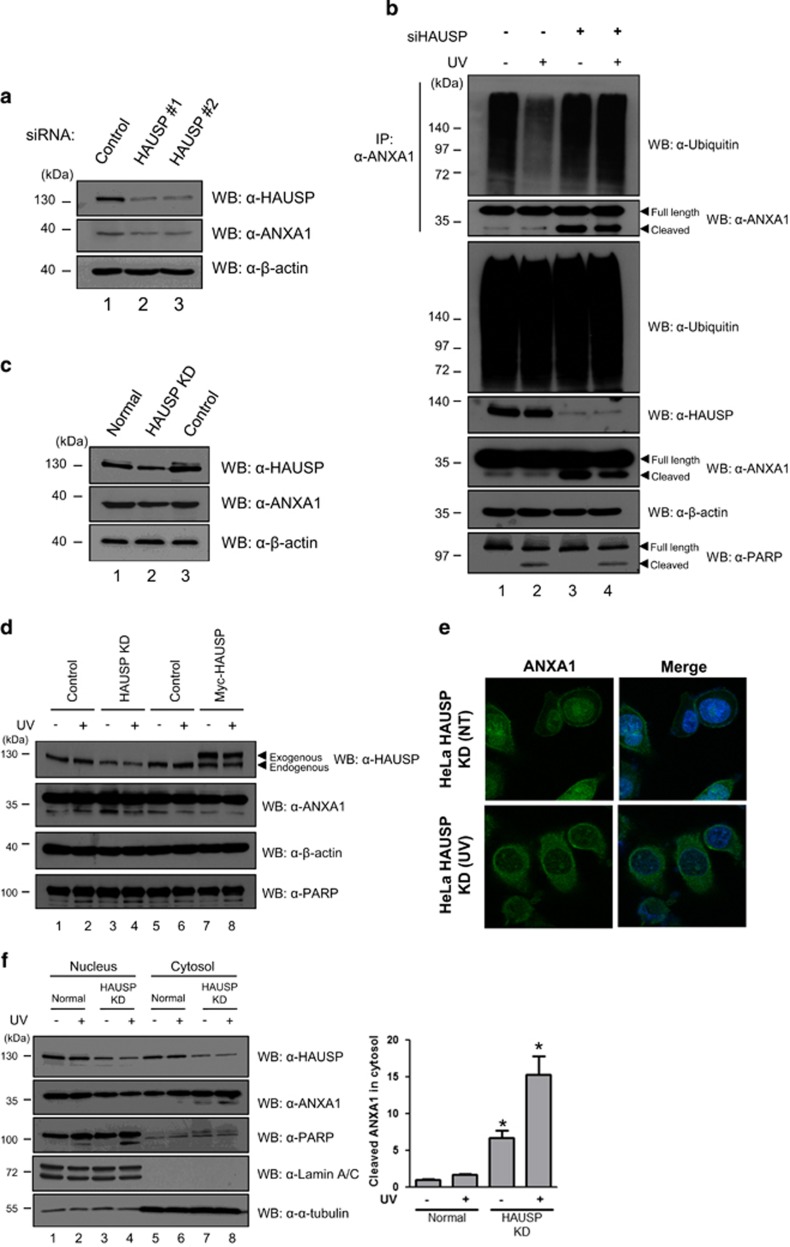
HAUSP knock-down effect on the regulation of ANXA1 following UV-induced DNA damage. (**a**) Two different HAUSP-specific siRNAs as described in *Materials and methods* were transfected into HeLa cells. The cells were harvested and lysed 72 h after incubation. The depleted HAUSP levels were detected by western blotting using an anti-HAUSP antibody. (**b**) HeLa cells transfected with siRNA specific for HAUSP or control siRNA were exposed to UV and incubated for 3 h. Then, cell lysates were immunoprecipitated with an anti-ANXA1 antibody. Subsequent western blotting was performed to detect the ubiquitination level of ANXA1 using an anti-ubiquitin antibody. (**c**) HAUSP-depleted (HAUSP KD) and control shRNA transduced (Control) HeLa cells were generated by lentiviral induction as described in *Materials and methods*. Depleted HAUSP levels compared with normal HeLa cells were detected by western blotting using an anti-HAUSP antibody. (**d**) HeLa, HAUSP knock-down HeLa (HAUSP KD), and HAUSP overexpressed HeLa cells either incubated for 3 h following UV treatment or not were blotted with anti-HAUSP and anti-ANXA1 antibodies to detect the cleavage of ANXA1 protein. (**e**) UV-treated or normal HeLa KD cells were immunostained using FITC staining after incubation with an anti-ANXA1 antibody. DAPI was used for counterstaining of the nucleus (Green: ANXA1, and Blue: DAPI). (**f**) Nucleus-cytosol fraction was performed using HeLa and HeLa KD cell lysates. Then, HAUSP and ANXA1 levels were detected using anti-HAUSP and anti-ANXA1 antibodies. Anti-PARP and anti-*α*-tubulin antibodies were used as fraction controls. Statistical data are presented as a means (*n*=3, *P<0.05)

We further investigated the functional effect of the interaction between HAUSP and ANXA1 following DNA damage. Because a previous study showed cleavage of ANXA1 upon various DNA damage including UV,^20^ we first confirmed ANXA1 protein levels in response to UV with the level of PARP cleavage as an UV irradiation control. As shown in Figure 4a, the cleaved form of ANXA1 gradually increased until 3 h after UV, and decreased cleaved ANXA1 was detected 4 h after UV in HeLa cells. The decrease of ANXA1 cleavage may be due to the DDR of UV-damaged cells.^26^ To further validate whether HAUSP is related to the cleavage of ANXA1, we performed endogenous IP assay in HeLa cells. The binding affinity of HAUSP to ANXA1 in a time-dependent manner following UV showed a gradual increase, and it peaked at 3 h after UV coinciding with the maximum of the cleaved ANXA1 (Figure 4b). On the basis of our result, we decided to incubate cells for 3 h following UV for further experiments. Interestingly, immunofluorescence analysis targeting endogenous HAUSP and ANXA1 in HeLa cells showed nuclear foci formation of both HAUSP and ANXA1 in the nucleus upon UV-induced DNA damage (Figure 4c), suggesting that HAUSP is possibly involved in the ANXA1-mediated damage response in the nucleus. Therefore, we validated the knock-down effect of HAUSP on the damage response function of ANXA1 using HAUSP-specific siRNA (Figure 5a). Then, we investigated the endogenous ubiquitination level of ANXA1 following UV in the presence or absence of HAUSP. The result showed that HAUSP could reduce the ANXA1 ubiquitination level more upon UV, possibly due to the increase in HAUSP-ANXA1 binding. However, in HAUSP-depleted cells, the ANXA1 ubiquitination level did not show any difference following UV (Figure 5b), indicating that HAUSP might be a specific DUB for ANXA1. Next, using a lentiviral vector that contains a HAUSP-specific shRNA, we generated HAUSP knock-down HeLa cells (Figure 5c). The level of cleaved form for ANXA1 has changed in HAUSP-depleted HeLa cells compared with the level found in normal HeLa cells. However, overexpression of HAUSP had no effect on ANXA1 cleavage upon UV exposure (Figure 5d). In addition, depletion of HAUSP affected the localization of ANXA1 (Figure 5e). Unlike normal HeLa cells, showing co-localization of HAUSP and ANXA1 in the nucleus by forming nuclear foci in response to UV, the absence of HAUSP abrogated the nuclear localization of ANXA1. We also confirmed the escape of ANXA1 from the nucleus following UV in the absence of HAUSP but not in the presence of HAUSP by fraction assay (Figure 5f). Collectively, our data suggest that the deubiquitination of ANXA1 is specifically mediated by HAUSP, and HAUSP-mediated deubiquitination of ANXA1 may be important for ANXA1's damage response function in the nucleus upon UV. In addition, the failure of deubiquitination of ANXA1 leads to cleavage of ANXA1, resulting in externalization of ANXA1 from the nucleus.

### Different roles of HAUSP by interacting with ANXA1 in Jurkat cells upon UV irradiation

**Figure 6:**
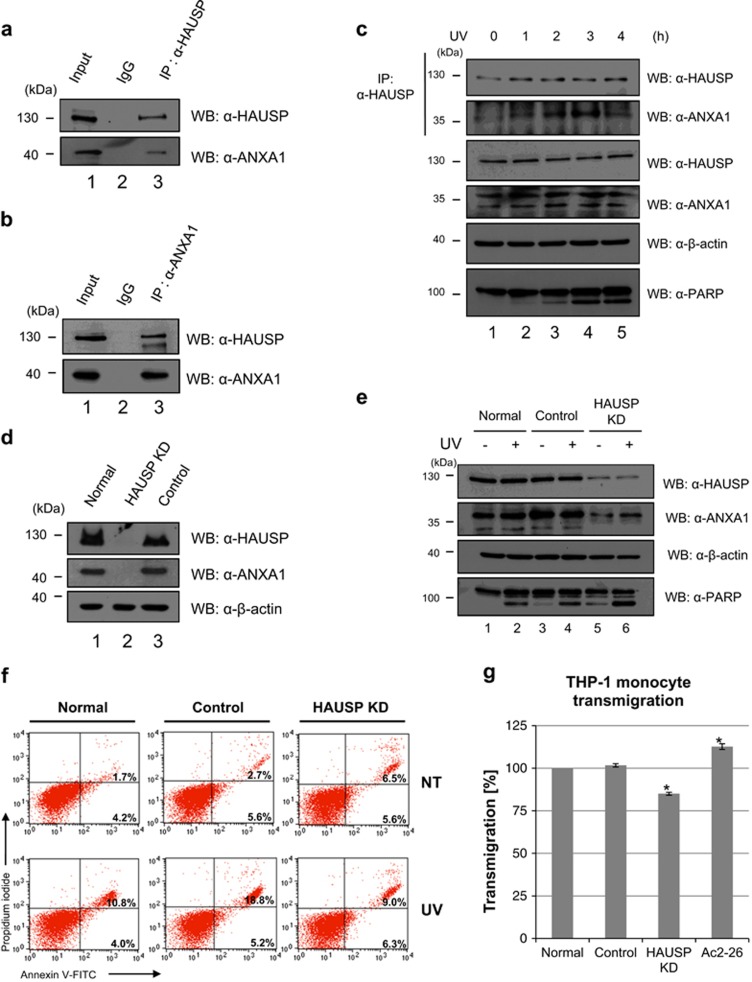
The role of interaction between HAUSP and ANXA1 following UV in Jurkat cells. (**a**, **b**) Endogenous binding of HAUSP and ANXA1 is confirmed by endogenous immunoprecipitation analysis. Jurkat cell lysates were immunoprecipitated with an anti-HAUSP or an anti-ANXA1 antibody. Then, western blotting was performed to detect ANXA1 or HAUSP expression using the respective antibodies. (**c**) Jurkat cells, time dependently incubated with 30 mJ/cm^2^ UV treatment, were lysed and immunoprecipitated with an anti-HAUSP antibody. Then, ANXA1 levels were detected by western blotting using an anti-ANXA1 antibody. (**d**) HAUSP-depleted (HAUSP KD) and control shRNA transduced (Control) Jurkat cells were generated as shown in Figure 4c. Depletion of HAUSP compared with normal Jurkat cells was detected by western blotting using an anti-HAUSP antibody. (**e**) Normal, control shRNA transduced (Control), and HAUSP-depleted Jurkat cells (HAUSP KD) were exposed to UV followed by incubation for 4 h. Then, cell lysates were immunoprecipitated with an anti-HAUSP antibody. Subsequent western blotting was performed with indicated antibodies. (**f**) Cells were stained with Annexin V-FITC/PI for verifying the apoptotic or necrotic cell ratio. (**g**) Jurkat, control, and HAUSP KD Jurkat cells were exposed to UV and incubated for 3 h. Then, cell supernatants were harvested and placed into the lower chamber. Subsequently, THP-1 monocytes cultured in serum-free media were placed into the upper chamber and incubated with respective cell supernatants in addition to Ac2-26 containing media. The transmigration ratio was detected as described in *Materials and Methods*. Each value represents the mean (±S.D.) of a representative of three independent experiments, and an asterisk mark (*) means statistical significance

It has previously been reported that ANXA1 itself can induce caspase-dependent apoptosis.^19, 27, 28, 29^ In addition, during secondary necrosis, the cleaved fragment of ANXA1 (Ac2-26) induces apoptotic cell clearance by recruiting macrophages.^20^ The study mainly evaluated ANXA1 cleavage, which is mediated by ADAM10 protease, during secondary necrosis using hematogenous cells including Jurkat cells, demonstrating that externalized Ac2-26 from apoptotic cells provokes macrophage activation for phagocytosis of dying cells. Therefore, we further validated the relevance of HAUSP with ANXA1 cleavage and subsequent functions in damaged cells using Jurkat cells. First, the binding between HAUSP and ANXA1 was confirmed via endogenous IP assay (Figures 6a and b). As expected, binding of HAUSP and ANXA1 was clearly detected. Intriguingly, their binding in Jurkat cells also time dependently increased 3 h after UV (Figure 6c). In addition, HAUSP had effect on the ubiquitination of ANXA1 in Jurkat cells (Supplementary Figures S6a and b). By generating HAUSP knock-down Jurkat cells (HAUSP KD) with a lentiviral shRNA for HAUSP (Figure 6d), we evaluated the importance of HAUSP in the regulation of ANXA1 upon UV-induced DNA damage. According to the results shown in HeLa cells, we considered the depletion of HAUSP to also affect ANXA1 cleavage in the Jurkat cells. However, the depletion of HAUSP reduced both the full-length and the cleaved form of ANXA1, contrary to increased cleavage of ANXA1 in HAUSP KD HeLa cells (Figure 6e). This unexpected result may be derived from multiple functions of ANXA1, which can be the main causes of different expression levels and roles of ANXA1 in different types of cancers.^30, 31, 32, 33^ Thus, we concluded that the failure of deubiquitination of ANXA1 by the depletion of HAUSP leads to reduced stability and cleavage of ANXA1 in the case of Jurkat cells, indicating that HAUSP is a positive regulator for ANXA1 in Jurkat cells. Using normal, control shRNA-transduced (control), and HAUSP KD Jurkat cells, we further evaluated the knock-down effect of HAUSP on apoptosis or necrosis level following UV. A previous study reported that the inhibition of the deubiquitinating activity of HAUSP using a small molecule that specifically inhibits HAUSP resulted in apoptosis in multiple myeloma (MM).^34^ We also detected increased late apoptotic/necrotic level in HAUSP KD Jurkat cells even in the absence of UV (Figure 6f). However, the ratio of the increase of late apoptotic/necrotic cell level following UV resulted in the lowest levels in HAUSP KD cells (Figure 6f; Supplementary Figure S7), suggesting that reduction of ANXA1 cleavage cannot increase apoptosis even after UV when HAUSP is depleted. Finally, we checked the transmigration of THP-1 monocytes toward cell supernatants derived from UV-treated normal, control, and HAUSP KD Jurkat cells. As expected, the transmigrative ratio of monocytes is the weakest in HAUSP-depleted cell supernatants (Figure 6g). Chemokines that attract migration of macrophage include various factors including Ac2-26. Thus, basal levels of transmigration ratio are shown even in HAUSP-depleted cells, and cell supernatants containing Ac2-26 showed increased transmigrative ratio. Overall, our findings demonstrate that depletion of HAUSP can decrease ANXA1 stability and cleavage of ANXA1, thereby reducing not only cell apoptosis upon UV but also transmigration of macrophages mediated by Ac2-26. These data suggest that deubiquitination of ANXA1 by HAUSP is essential for the UV-induced damage response function of ANXA1 in Jurkat cells.

## Discussion

Numerous studies have demonstrated that the expression patterns of ANXA1 in cancers vary. For example, downregulated expression of ANXA1 is shown in thyroid cancer, prostate cancer, head and neck cancer, esophageal cancer, hairy cell leukemia (HCL), and B-cell non-Hodgkin's lymphomas.^35, 36, 37, 38, 39, 40^ However, it is also reported that ANXA1 is overexpressed in hepatocarcinoma and pancreatic cancer.^41, 42^ Moreover, in breast cancer, the expression of ANXA1 may be dependent on specific subtypes. de Graauw *et al.*^43^ validated the mRNA levels of ANXA1 in both luminal- and basal-like breast cancer cells, and the results showed upregulated mRNA levels of ANXA1 in basal-like breast cancer cells whereas luminal-like breast cancer cells showed reduced mRNA levels of ANXA1.^43^ These different expression patterns of ANXA1 highlight that ANXA1 may be regulated by different mechanisms. Indeed, it is well known that ANXA1 is regulated via various posttranslational modifications. Ubiquitination of ANXA1 mediated by E6-associated protein (E6AP) has been reported previously.^25^ We could also demonstrate ubiquitination and deubiquitination of ANXA1 (Figures 3a and b). HAUSP reduced the level of ANXA1; however, a catalytically inactive mutant of HAUSP (C223S) has less effect on the ANXA1 ubiquitination (Figure 3b), which is different from the fully denatured condition (Figure 3d). Because the non-catalytic function of DUBs was reported,^44^ it is presumed that the catalytic inactive mutant of HAUSP also has effect on ANXA1 ubiquitination through the interaction of other DUBs or E3 ligases *in vivo*, and further biochemical studies to understand this subtlety are required. In addition to ubiquitination, two recent different studies have investigated the modification of ANXA1 through small ubiquitin-like modifiers (SUMO) in a mouse model.^45, 46^ In particular, it showed that mammalian ANXA1 has the consensus motif ^160^LKRD for SUMOylation, suggesting that human ANXA1 can also be modified by SUMO. ^46^ Because SUMOylation can affect protein-protein interaction or protein localization,^47^ it is also possible that SUMOylated ANXA1 may have altered localizations or functions. The alterative localization of ANXA1 by post-translational modification is demonstrated by Solito *et al.*^48^ They found the translocation of cytosolic ANXA1 to the cell surface via phosphorylation at serine-27 in lipopolysaccharide (LPS)-treated cells. In this regard, our result of immunofluorescence analysis in HAUSP KD HeLa cells, which showed translocation of nuclear ANXA1 to the cytosol (Figure 5e), may be due to the failure of deubiquitination of ANXA1 mediated by HAUSP.

**Figure 7:**
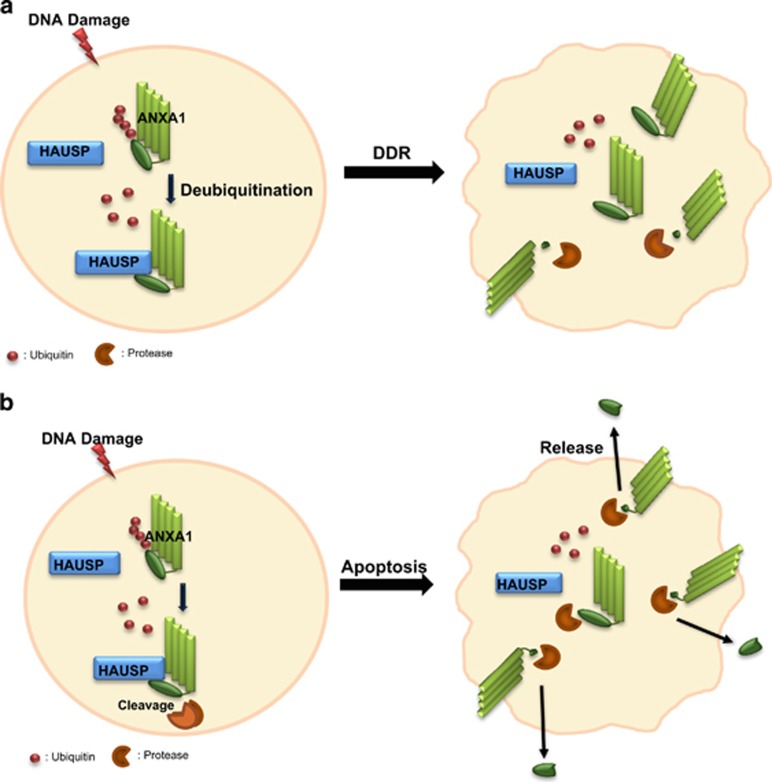
A schematic model for UV damage response by the interaction between HAUSP and ANXA1. (**a**) UV damage response of HAUSP-ANXA1 in HeLa cells. HAUSP deubiquitinates ANXA1 and protects ANXA1 from the proteasomal degradation and protease-mediated cleavage, leading to stress response function of ANXA1 upon UV damage. (**b**) UV damage response of HAUSP-ANXA1 in Jurkat cells. HAUSP mediates ANXA1 cleavage, leading to dying cell clearance function of ANXA1 upon UV damage

Nuclear localization of ANXA1 has been broadly studied for several years. Over 50% of oral squamous cell carcinoma (OSCC) showed nuclear localization of ANXA1,^49^ suggesting that nuclear ANXA1 can be a prognostic factor in OSCC. Na *et al.* suggested the role of ANXA1 in cellular stress condition.^18^ They showed that ANXA1 is located in the nucleus by the induction of stress reagents including heat, oxidative stress, and a sulfhydryl-reactive agent. Heat-shock damage also caused nuclear translocation of ANXA1 in MCF7 breast cancer cells, resulting in protection of cancer cells from the damage via unknown mechanism.^50^ Finally, Yan *et al.*^22^ analyzed ANXA1 as one of nuclear proteins of BaP-treated HeLa cells by using the 2-DE method. They suggested that ANXA1 may have a protective role in BaP-induced damage response. We also showed nuclear ANXA1 in HeLa cells (Figure 2e). Interestingly, we could detect nuclear localization of ANXA1 forming nuclear foci together with HAUSP when UV was inflicted on HeLa cells (Figure 4c). It is noteworthy that UV induces nuclear foci formation, leading to the induction of DDR.^51, 52^ Thus, our data for the failure of foci formation of ANXA1 in HAUSP-depleted HeLa cells (Figure 5e) indicate that HAUSP may be required for the DDR function of ANXA1 following UV-induced DNA damage in certain types of cells (Figure 7a).

It is notable that, in contrast to HeLa cells, reduced full-length and cleavage of ANXA1 in HAUSP knockdown condition was detected in Jurkat cells (Figure 6e). As previously reported, overexpression of ANXA1 can lead to cell apoptosis.^19, 27, 28, 29^ Our result also demonstrated that reduced ANXA1 by the absence of HAUSP shows reduced apoptotic level after exposure to UV (Figure 6f). The cleavage of ANXA1 is well established in immune regulation. Blume *et al.*^20^ demonstrated that ADAM10 specifically cleaved ANXA1, and Ac2-26 provided a ‘find-me' signal to monocytes for dying cell clearance, functioning as a chemokine. Although there are various chemotaxic components, our data showed that even the absence of only Ac2-26, resulting from HAUSP depletion, could reduce the transmigration of THP-1 monocytes toward dying cells (Figure 6g), indicating that HAUSP is partially related to the dying cell clearance through the regulation of ANXA1 cleavage (Figure 7b).

In conclusion, we showed a line of evidence that ANXA1 is regulated by the deubiquitinating activity of HAUSP, and the following results vary depending on cell types. Altered binding affinity toward HAUSP, localization, and functional cleavage of ANXA1 were shown in the absence of HAUSP following UV. In this regard, we suggest that deubiquitination of ANXA1 by HAUSP is essential for UV-induced damage responsive function of ANXA1. To evaluate the relevance of HAUSP-ANXA1 interaction with damage response function as shown in our results of HeLa cells, further mechanism studies of signaling pathway should be validated. Similarly, it would be of interest to establish the details underlining the deubiquitination and cleavage of ANXA1 in the resolution of the immune system to provide insights into the molecular regulation of ANXA1 by HAUSP.

## Materials and Methods

### Cell culture and UV induction

HeLa, human embryonic kidney (HEK) 293T, and 293FT cells were grown in Dulbercco's modified Eagle's medium (DMEM, Gibco, Grand Island, NY, USA) supplemented with 10% FBS, 1% penicillin/streptomycin (Gibco). Jurkat T and THP-1 cells were grown in RPMI-1640 medium supplemented with 10% FBS, 1% penicillin/streptomycin (Gibco). Cells were grown at 37 °C in 5% CO_2_ atmosphere. To evaluate damage response mediated by UV, cells were UV irradiated with 30 mJ/cm^2^ in the UV cross-linker (CL-1000 UV crosslinker, UVP, CA, USA) for the indicated time.

### Expression constructs and antibodies

A full-length cDNA for human HAUSP, ANXA1, PKM2, and PP2A was PCR amplified from HeLa and subcloned into the Flag, Myc, and GST epitope encoded vectors, respectively. Following antibodies were used for western blotting or IP: anti-Myc (9E10, Hybridoma cell media), anti-HA (1:200, 12CA5 hybridoma cell media), anti-Flag (1:5000 Sigma-Aldrich, St. Louis, MO, USA), anti-HAUSP (1:1000, Santa Cruz Biotechnology, Santa Cruz, CA, USA), anti-ANXA1 (1:1000, BD Bioscience, San Jose, CA, USA or 1:000, Young In Frontier, Seoul, South Korea), anti-PARP1 (1:1000, Santa Cruz Biotechnology), anti-*β*-actin (1:3000, Santa Cruz Biotechnology), anti-*α*-tubulin (1:1000, Santa Cruz Biotechnology) and anti-ubiquitin (1:1000, Santa Cruz Biotechnology) antibody.

### Site-direct mutagenesis

A catalytic mutant of HAUSP (C223S) and ANXA1 mutants (S5A, S182A, S5A/S182A) were generated by site-direct mutagenesis. The following primer sequences were used for generating mutants: ANXA1 (S5A); forward, 5′-ATG GCA ATG GTA GCA GAA TTC CTC A-3′, reverse, 5′-TGA GGA ATT CTG CTA CCA TTG CCA T-3′, ANXA1 (S182A); forward, 5′-CGG AAC GCT TTG CTT GCT CTT GCT AAG GGT G-3′, reverse, 5′-CAC CCT TAG CAA GAG CAA GCA AAG CGT TCC G-3′. ANXA1 mutant (S5A/S182A) was generated using ANXA1 (S5A) as a template and ANXA1 (S182A) primer. After PCR and gel purification, Dpn I (Enzynomics, Daejeon, Korea) enzyme was added.

### RNAi interference and knock-down cell lines using lentiviral vectors

siRNAs for HAUSP were generated with following sequences: #1; 5'-CAU GCA CAA GCA GUG CUG AAG AUA A-3', #2 5′-AAA GU U UCC CAC CCA AAU GAC UUU G-3′ (Ambion, Austin, TX, USA). In all, 20 nM of HAUSP siRNA was introduced into the cells by transfection using Opti-MEM and RNAimax (Invitrogen, Paisly, UK) according to the manufacturer's instructions. For the generation of stably HAUSP-depleted, HAUSP knock-down cells (HAUSP KD) or control shRNA transduced cells (Control), lentiviral shRNAs with packaging DNA vectors were transfected into HEK 293FT cells using Lipofectamine 2000 (Invitrogen). Forty-eight hours after transfection, the cell supernatants were filtrated. Then, the lentivirus contained in the supernatants was transduced into HeLa or Jurkat cells together with polybrene (Sigma-Aldrich). Finally, puromycin (Sigma-Aldrich) for the selection of shRNA-transduced cells was used during cell culture.

### Western blotting, IP, GST-pull down assay, ubiquitination, and deubiquitination assays

For western blotting, IP, ubiquitination, and deubiquitination assays, cells were lysed in a lysis buffer (150 mM NaCl, 50 mM Tris-HCl (pH 7.4), 1 mM PMSF, 1 mM DTT, 1% NP-40 and protease inhibitor cocktail (PIC) (Roche, Mannheim, Germany)). After resuspension of cells, samples were incubated for 20 min on ice and centrifuged at 13 000 r.p.m. for 15 min. Then, proteins were loaded onto 10% SDS-PAGE gel and transfered onto polyvinylidene fluoride (PVDF) microporous membranes (Millipore, Billerica, MA, USA). Membranes were blocked with 5% skim milk in TBST (20 mM Tris-HCl (pH 7.5), 150 mM NaCl) for 1 h and incubated with primary antibodies at 4 °C overnight. The membranes were incubated with secondary antibodies followed by detection using the ECL reagent solution (Young In Frontier). Membranes were stripped with stripping buffer (Amresco, Solon, OH, USA) according to the manufacture's protocol. For co-IP assay, cell lysates were incubated with indicated antibodies for 4 h at 4 °C. Then, protein A/G PLUS agarose beads (Santa Cruz Biotechnology) were added and rotated for 1 h. The precipitated protein complexes were blotted by western blotting. GST-pull down assay was performed as previously described.^53^ Ubiquitination and deubiquitination assays were performed using HA-tagged ubiquitin-transfected cell lysates. After IP with an anti-HA antibody, protein samples were analyzed by western blotting. Ubiquitination bands were detected using an anti-HA antibody. Western blotting images were analyzed with Image J program (National Institutes of Health (NIH), Bethesda, MD, USA).

### His-tagged protein purification with Ni-NTA beads

HEK 293T cells transfected with His-tagged ubiquitn were lysed with urea lysis buffer (8 M urea, 100 mM NaPO_4_ (pH 8.0), 15 mM imidazole). After sonication, the samples were incubated at room temperature and centrifuged for 20 min at 14 000 r.p.m. at 10 °C. Cell extracts were incubated with Ni-NTA beads (Novagen, Madison, WI, USA) at room temperature. Beads were washed with washing buffer (6 M GuHCl, 0.1% Triton X-100, 100 mM NaPO_4_ (pH 8.0), 15 mM imidazole) two times, and once with urea lysis buffer containing 0.1% Triton X-100, and finally washed with urea lysis buffer. For protein elution, beads were eluted with elution buffer (8 M urea, 100 mM NaPO_4_ (pH 8.0), 100 mM imidazole) for 5 min at room temperature.

### Immunofluorescence analysis

HeLa and Jurkat T cells (5 × 10^3^ or 1 × 10^4^ per 12 mm flame sterilized coverslips) treated with UV (30 mJ/cm^2^) or without were washed with 1 × PBS and fixed with 5% formaldehyde (Sigma-Aldrich) for 10 min at room temperature. Then, cells were rinsed three times with 0.1% Triton X-100/PBS, and blocked with 3% skim milk in 0.1% Triton X-100/PBS for 1 h at room temperature. The cells were incubated with anti-HAUSP and/or anti-ANXA1 antibodies for 2 h at room temperature. After, the cells were washed three times with 0.1% Triton X-100/PBS, and then incubated with 1:200 diluted FITC-conjugated goat anti-rabbit or anti-mouse IgG (Molecular Probes, Eugene, OR, USA). Cells were stained with 4', 6-diamindino-2-phenylindole (DAPI) (Molecular Probes) for nuclei staining. Afterward, coverslips were mounted onto glass slides using 90% glycerol/100 mM Tris (pH 8.0) and cell images were captured by using a Zeiss Axiovert 100 M microscope (Carl Zeiss, Oberkochen, Germany) attached to an LSM 510 confocal unit.

### Fraction assay

For the nucleus and cytosol fraction, resuspension buffer (10 mM HEPES, 10 mM KCl, 1.5 M MgCl_2_, and protease inhibitor cocktail) with 0.1% of NP-40 was used. Fifteen minutes after ice incubation, cell lysates were centrifugated at 14 000 r.p.m. for 2 min at 4 °C. The supernatants were for cytosolic fraction, and the pellet was washed with resuspension buffer without NP-40. Then, TNN buffer (50 mM Tris, 250 mM NaCl, 5 mM EDTA, and protease inhibitor cocktail) with 0.5% of NP-40 was used for pellet resuspesion. After incubation on ice for 1 h, samples were homogenized with 1 ml syringe and centrifugated at 16 000 r.p.m. at 4 °C for 40 min. Finally, the supernatants were used as nuclear fraction.

### Flow cytometry

Flow cytometry was performed with a FACS Calibur (BD Bioscience) and CellQuest analysis software. Apoptosis was determined by staining with Annexin V-FITC/propidium iodide (PI) (BD BioScience) double staining according to the manufacturer's instructions. Briefly, cells (2 × 10^6^) were washed with cold PBS and then resuspended in binding buffer (10 mM Hepes/NaOH (pH 7.4), 0.14 M NaCl, 2.5 mM CaCl_2_), and FITC Annexin V and PI were added. After incubation at room temperature for 15 min in the dark, flow cytometry was analyzed. Annexin V-FITC and PI double staining were regarded as late apoptotic or necrotic cells.

### Transmigration assay

Transmigration assay was performed using the QCM Chemotaxis migration assay kit (Millipore) according to the manufacturer's instruction. Briefly, THP-1 (1 × 10^5^) cells were incubated in the upperside of membranes, and bottom wells were filled with cell-culture supernatants or medium or the N-terminal fragment of ANXA1 (Ac2-26). Ten hours after incubation, cells were lysed with a lysis buffer with the CyQuant GR dye. Then, fluorescence was detected using 480/520 filter set. The percentage of THP-1 monocyte migration was calculated by regarding serum containing normal cell supernatants as a hundred percent.

### 2-DE and MALDI-TOF/MS analyses

Aliquots in sample buffer (7 M urea, 2 M thiourea, 4.5% CHAPS, 100 mM DTE, 40 mM Tris, pH 8.8) were applied to immobilized pH 3–10 non-linear gradient strips (Amersham Biosciences, Uppsala, Sweden). IEF was performed at 80 000 Vh. The second dimension was analyzed on 9–16% linear gradient polyacrylamide gels (18 cm 6 20 cm 6 1.5 mm) at constant 40 mA per gel for approximately 5 h. After protein fixation in 40% methanol and 5% phosphoric acid for 1 h, the gels were stained with CBB G-250 for 12 h. The gels were destained with H_2_O, scanned in a Bio-Rad GS710 densitometer (Richmond, CA, USA) and converted into electronic files, which were then analyzed with Image Master Platinum 5.0 image analysis program (Amersham Biosciences). Spots were considered to be reproducibly present in all 2-DE gels, which samples were prepared from three independent HAUSP-overexpressing cell extracts.

For MALDI-TOF/MS analysis, the peptides were concentrated by a POROS R2, Oligo R3 column (Applied Biosystems, Foster City, CA, USA). After washing the column with 70% acetonitrile, 100% acetonitrile and then 50 mM ammonium bicarbonate, samples were applied to the R2, R3 column and eluted with cyano-4-hydroxycinamic acid (CHCA) (Sigma) dissolved in 70% acetonitrile and 2% formic acid onto the MALDI plate (Opti-TOFTM 384-well Insert, Applied Biosystems). MALDI-TOF/MS was performed on 4800 MALDI-TOF/TOF Analyzer (Applied Biosystems) equipped with a 355-nm Nd:YAG laser. The pressure in the TOF analyzer is approximately 7.6e-07 Torr. The mass spectra were obtained in the reflection mode with an accelerating voltage of 20 kV and sum from either 500 laser pulses and calibrated using the 4700 calibration mixture (Applied Biosystems). NCBInr database (downloaded on Mar 242,013, # of entries=695 124 sequence) searching was performed with the MASCOT search engine (http://www.matrixscience.com). The peak list-generating software Data explorer version 4.4 (PerSeptiveBiosystems, Framingham, MA, USA) was used. Database search criteria were taxonomy, Homo sapiens, fixed modification; carboxyamidomethylated (+57) at cysteine residue; variable modification; oxidized (+16) at methionine residues, maximum allowed missed cleavage, 1, MS tolerance, 100 ppm. Only peptides resulting from trypsin digests were considered, and trypsin peak (842.5090, 2211.1040) was used for internal calibration.

## Figures and Tables

**Table 1:** Protein identities determined by MALDI-TOF/MS

**Spot ID**	**Accession number**	**Protein name**	**Score**^a^	**Matched peptide number**	**Sequence coverage (%)**	**Matching sequence**	**Theoretical MW/pI**
33	gi|31645	Glyceraldehyde-3-phosphatedehydrogenase	108	14/45 (31%)	39	LVINGNPITIFQERDPSKIKWGDAGAEYVVESTGVFTTMEK, RVIISAPSADAPMFVMGVNHEK, GALQNIIPASTGAAK, V IPELDGKLTG MAFRVPTANV SVVDLTCR, L ISWYDNEFGY SNRVVDLMAH MASK	36202/8.26
36	gi|9367836	FOG2, friend of GATA2, altenatively spliced product	71	10/124 (8%)	63	TKAQVPMVLTAGPKWLLDVTWQGVEDNKNNCIVYSKGGQLWCTTTKAISEGEELIALVVDF DSRLQAASQMTLTEGMYPAR, CSVLCSPALEVMGIYGRK	17185/9.13
181	gi|189306	Nucleolin	126	13/26 (50%)	23	TGISDVFAKNDLAVVDVR, VTQDELKEVFEDAAEIR, SKGIAYIEFK, SISLYYTGEKGQNQDYR, TLVLSNLSYSATEETLQEVFEK, SKGYAFIEFASFEDAK, AIRLELQGPR, GLSEDTTEETLKESFDGSVR, GFGFVDFNSEEDAKEAMEDGEIDGNKVTLDWAKPK	76355/4.59
255	gi|83699649	Heat-shock 90 kDa protein 1, alpha	77	16/66 (24%)	20	DRLDPRPGSPSEASSPPFLR, ELISNSSDALDKIR, HNDDEQYAWESSAGGSFTVRTDTGEPMGRGTKVILHLKEDQTEYLEER, HSQFIGYPITLFVEKER, SLTNDWEDHLAVKHFSVEGQLEFR, RAPFDLFENR, GVVDSEDLPLNISR	98082/5.07
323	gi|46249758	Villin 2 (ezrin)	76	11/25 (44%)	19	VTTMDAELEFAIQPNTTGK, LFFLQVK, DQWEDRIQVWHAEHR, IAQDLEMYGINYFEIK, FVIKPIDKKAPDFVFYAPR, SQEQLAAELAEYTAK, QLLTLSSELSQAR	69313/5.94
395	gi|5729877	Heat-shock 70 kDa protein 8 isoform 1	137	11/14 (78%)	22	TTPSYVAFTDTER, HWPFMVVNDAGRPK, TVTNAVVTVPAYFNDSQR, DAGTIAGLNVLRIINEPTAAAIAYGLDKK, MVNHFIAEFK, ARFEELNADLFR, LDKSQIHDIVLVGGS TR, QTQTFTTYSDNQPGVLIQVYEGER, NSLESYAFNMK	71082/5.37
550	gi|33870117	PKM2 protein 1	127	14/26 (53%)	29	LDIDSPPITAR, LNFSHGTHEYHAETIK, TATESFASDPILYRPVAVALDTKGPEIR, IYVDDGLISLQVK, FGVEQDVDMVFASFIR, RFDEILEASDGIMVAR, MQHLIAREAEAAIYHLQLFEELRRLAPITSDPTEATAVGAVEASFK, GHVVIVLTGWRPGSGFTNTMR	62046/8.99
552	gi|4680681	CGI-21 protein	64	10/71 (14%)	36	SSIPHWRISRMCLKPTFTK, AYDGTTYLPGIVGLNNIK, RPPGDIMFLLVQR, AHVSPHEMLQAVVLCSKK, KLPHPDLPAEEK, EQLIIPQVPLFNILAK, LPPYLIFCIKIFTKNNFFVEK	37518/9.47
605	gi|119570522	Interferon-induced protein with tetratricopeptide repeats 1, isoform CRA_b	66	12/70 (17%)	34	VLDQIEFLDTK, FRYRMECPEIDCEEGWALLKCGGK, VLEVDPENPESSAGYAISAYR, NHKPFSLLPLR, YIEEALANMSSQTYVFR, SAIFHFESAVEKKPTFEVAHLDLAR, KSDVNAIIHYLKAIKIEQASLTR, LEGNMNEALEYYE R, LAADFE NSVR	51681/8.55
608	gi|73620945	Bullous pemphigoid antigen 1, isoforms 6/9/10 (Trabeculin-beta)	73	41/81 (50%)	9	VQRDSVICEDK, NECSSVYSKGR, AIEEFESSLKEAK, ELDQKEENIK, EELLQYKSTIANLMGKTIIQLKPR, SVVSWHYLINEIDR, ISSEEISTKK, QQEYKEK, GDLRYITISGNR, LDHATDRFR, HLSEPIAVDPK, QSSINAMNEKVK, EAVTSCQEQLDAFQVLVK, AQEESSAMMQWLQK, SFEAELKQNVNK, WKQMLTEIDSK, CSFLETKLQGIGHFR, MMLATEETSPDLVGIK, LSVQDYSTEGLWKQQSELR, TLEQALQLAR, ALLDSLNEVSSALLELVPWR, LEQDQTSAQQVQKTFTMEILRHR, NYDTICQINSER, LQPLYETLK, FWCDHMSLIVTIK, QQIEELK, HHVLQNDVLAHQSTVEAVNK, WQNVLEKTEQR, SAETNIDQDINNLK, SLGAKHSVYDTTNR, DDSSWVKVQMQELSTR, ARFEEVLAWAKQELLEALLAWLQWAETTLTDK, DAYKPITDADKIEDEVTRQVAK, FQVEQIGDNKYR	590626/5.49
609	gi|5174735	Tubulin, beta, 2	121	15/65 (23%)	41	FWEVISDEHGI DPTGTYHGDSDLQLER, AVLVDLEPGTMDSVRSGPFGQIFRPDNFVFGQSGAGNNWAKGHYTEGAELVDSVLDVVRK, IMNTFSVVPSPK, FPGQLNADLRKLAVNMVPFPR, ALTVPELTQQMFDAK, YLTVAAVFR, NSSYFVEWIPNNVK, MSATFIGNSTAIQELFKRISEQFTAMFR	49799/4.79
1081	gi|4502101	Annexin I	71	13/99 (13%)	52	QAWFIENEEQEYVQTVK, GGPGSAVSPYPTFNPSSDVAALHK, GVDEATIIDILTKR, AAYLQETGKPLDETLKKALTGHLEEVVLALLKTPAQFDADELR, GLGTDEDTLIEILASR, KGTDVNVFNTILTTR, CATSKPAFFAEKLHQAMK, SEIDMNDIKAFYQKMYGISLCQAILDETKGDYEK	38690/6.57
1117	gi|48146951	PPP2CA	67	10/86 (11%)	47	EILTKESNVQEVR, SPDTNYLFMGDYVDRGYYSVETVTLLVALK, ALDRPQEVPHEGPMCDLLWSDPDDR, GAGYTFGQDISETFNHANGLTLVSRAHQLVMEGYNWCHDRNVVTIFSAPNYCYR, YSFLQFDPAPR, GEPHVTRRTPDYFL	35527/5.30
1223	gi|62897717	Lactate dehydrogenase A variant	80	11/67 (16%)	28	MATLKDQLIYNLLKEEQTPQNK, DLADELALVDVIEDKLKGEMMDLQHGSLFLR, LVIITAGAR, FIIPNVVK, EVHKQVVESAYEVIK, KSADTLWGIQK	36666/7.63
1781	gi|55957383	Zinc finger protein 618	57	19/120 (15%)	23	NQPGGAAAPQADGASAAGRK, NQQTLDGKAPEGSPHGGSVR, YASGSYECGICGKK, TQTNQSGKK, CATLLHRTPPATQTQTFRTPNSGSPASK, SPPAVVEEK, NSANNTTTSGLTPNSMIPEK, VSTSAFSKAGMCLRCSACALNSVVQSVLSK, LCHLFLEALK, VAMILDPQQK, KPRSAAVENPAAQEDDR, LAFWLLAVPAVGAR, RLLSPEDMNK	104889/6.70
2014	gi|4504301	H4 histone family, member A	110	9/26 (34%)	56	DNIQGITKPAIRR, ISGLIYEETRGVLKVFLENVIRDAVTYTEHAK, TVTAMDVVYALKR	11360/11.36

a Score is −10 × Log(*P*), where *P* is the probability that the observed match is a random event; it is based on NCBInr database using the MASCOT searching program as MS/MS data.

## Abbreviations

DDR: DNA damage response
DUBs: deubiquitinating enzymes
E6AP: E6-associated protein
HCL: hairy cell leukemia
JAMM: JAB1/MPN/Mov34 metalloenzyme
MCPIPs: monocyte chemotactic protein-induced proteases
MM: multiple myeloma
MJDs: Machado-Josephin domain papain-like cysteine proteases
OSCC: oral squamous cell carcinoma
OUT: ovarian tumor
SUMO: small ubiquitin-like modifiers
UCHs: ubiquitin C-terminal hydrolases
UPP: ubiquitin-proteasome pathway
USPs: ubiquitin-specific proteases
