# Supplementary material for: Annexin-1 regulated by HAUSP is essential for UV-induced damage response
Source: Cell Death Dis. 2015 Feb 19;6(2):e1654–. doi: 10.1038/cddis.2015.32 (PMC4669820; doi:10.1038/cddis.2015.32)
Supplement: Supplementary Figure legends [file cddis201532x1.doc]

**Supplemental data figure legends**

**Figure S1.** **2-DE gel analysis of HAUSP in HeLa cells.** HeLa cells expressing Myc-tagged HAUSP were lysed and these cell extracts were fractionated on 2-DE gels. Samples were subjected to immunoblotting analysis with anti-Myc and anti-α-tubulin antibodies.

**Figure S2**. **Results for MS spectra of all detected.** Mass peaks are shown with MS analysis of the peptides from the 2-DE gel, which were prepared by in-gel digestion using trypsin. Trypsin peaks (842.5090, 2211.1040) were used for internal calibration.

**Figure S3.** **HAUSP interacting proteins undergo post-translational modification.** The cDNA from Myc-tagged HAUSP and mock vector transfected cells as a control was obtained by PCR using appropriate primers.

**Figure S4.** **HAUSP interacts ANXA1 in vitro.** **(a)** Purified GST and GST-tagged HAUSP proteins were incubated with cell lysates from HEK 293T cells overexpressed with Flag-ANXA1 (CBB, Coomassie brilliant blue). After incubation, each protein complex was analyzed by Western blotting with indicated antibodies. **(b)** Bands showing GST-tagged HAUSP was selected and analyzed by MALDI-TOF-MS analysis.

**Figure S5.** **HAUSP increases the expression level of ANXA1.** HEK 293T cells were transfected with Myc-HAUSP in a dose dependent manner and Flag-ANXA1. Exogenous HAUSP and ANXA1 levels were detected by Western blotting using anti-Myc and anti-Flag antibodies. Statistical data are presented as a means (n=3, *p<0.05).

**Figure S6.** **The deubiquitinating enzyme activity of HAUSP on ANXA1 in Jurkat cells.** **(a)** Cell lysates from Jurkat cells transfected with Myc-HAUSP or a catalytically inactive form of Myc-HAUSP (C223S), along with Flag-ANXA1 and HA-ubiquitin were immunoprecipitated with an anti-Flag antibody. Subsequently, Western blotting was performed to detect the ubiquitination level of ANXA1. **(b)** Cell lysates from Jurkat cells transfected with siRNA specific for HAUSP or control siRNA, along with Flag-ANXA1 and HA-ubiquitin, were immunoprecipitated with an anti-Flag antibody. Subsequent Western blotting was performed to detect the ubiquitination level of ANXA1 using an anti-HA antibody.

**Figure S7. UV-induced apoptosis of Jurkat cells.** Jurkat cells non-treated or treated with UV for 6 h were stained with propidium iodide, and the sub-G1 (M) peak as an apoptotic population was determined with FACS analysis.
