# Supplementary material for: Annexin-1 regulated by HAUSP is essential for UV-induced damage response
Source: Cell Death Dis. 2015 Feb 19;6(2):e1654–. doi: 10.1038/cddis.2015.32 (PMC4669820; doi:10.1038/cddis.2015.32)
Supplement: Supplementary Figures [file cddis201532x2.ppt]

## Slide 1
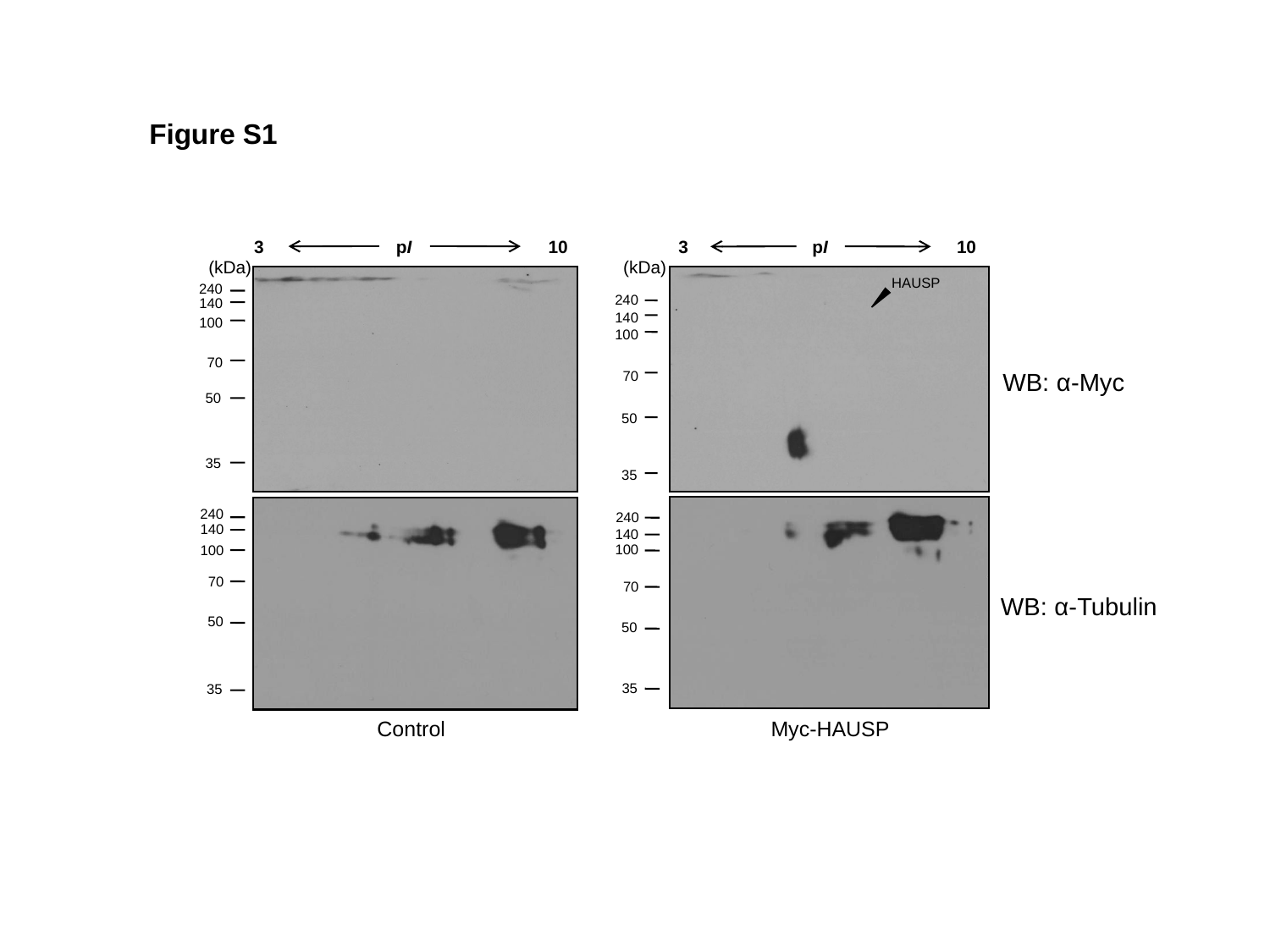

Figure S1
pI
3
10
pI
3
10
(kDa)
(kDa)
HAUSP
240
240
140
140
100
100
70
70
WB: α-Myc
50
50
35
35
240
240
140
140
100
100
70
70
WB: α-Tubulin
50
50
35
35
Control
Myc-HAUSP

## Slide 2
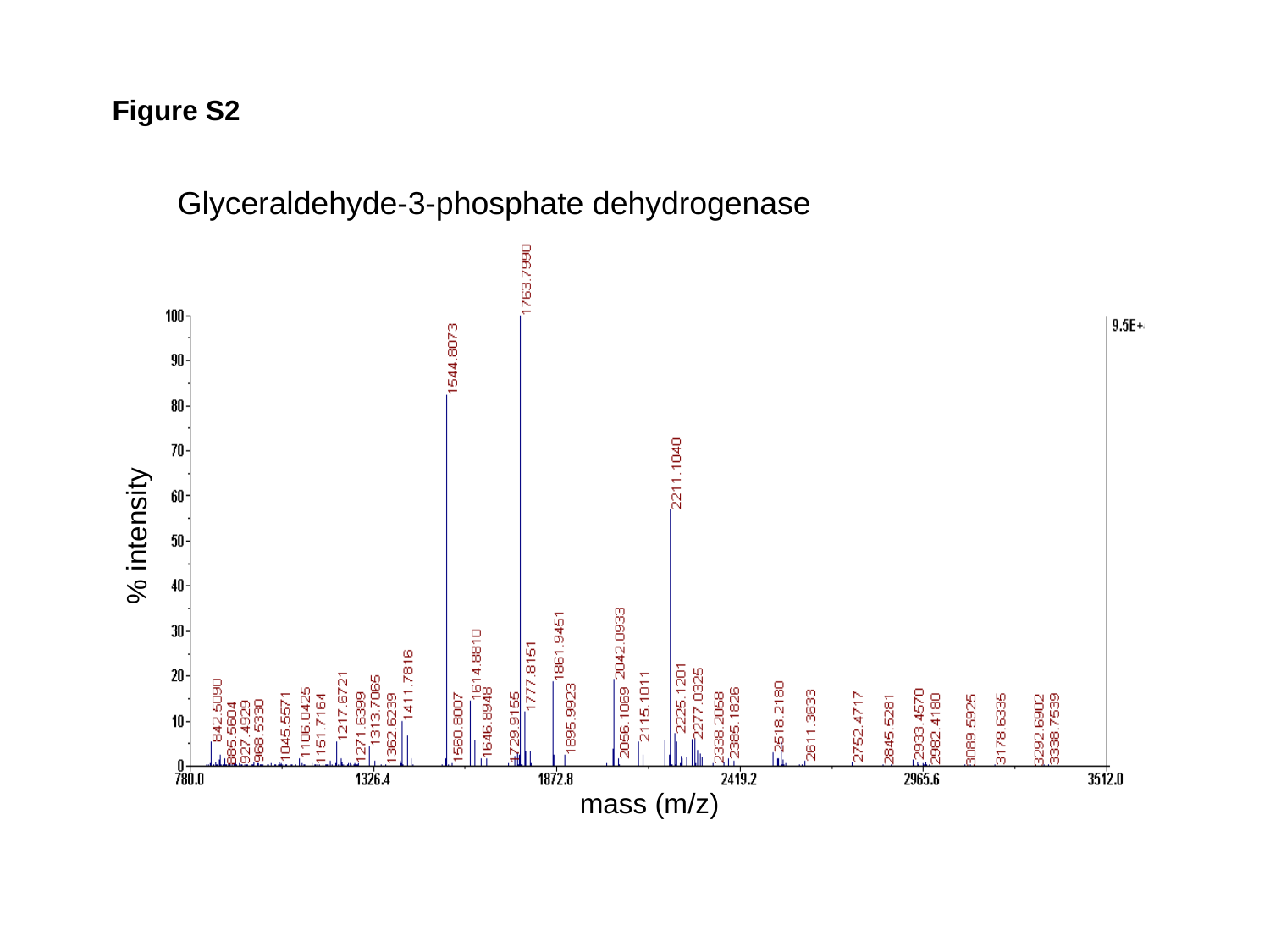

Figure S2
Glyceraldehyde-3-phosphate dehydrogenase
% intensity
mass (m/z)

## Slide 3
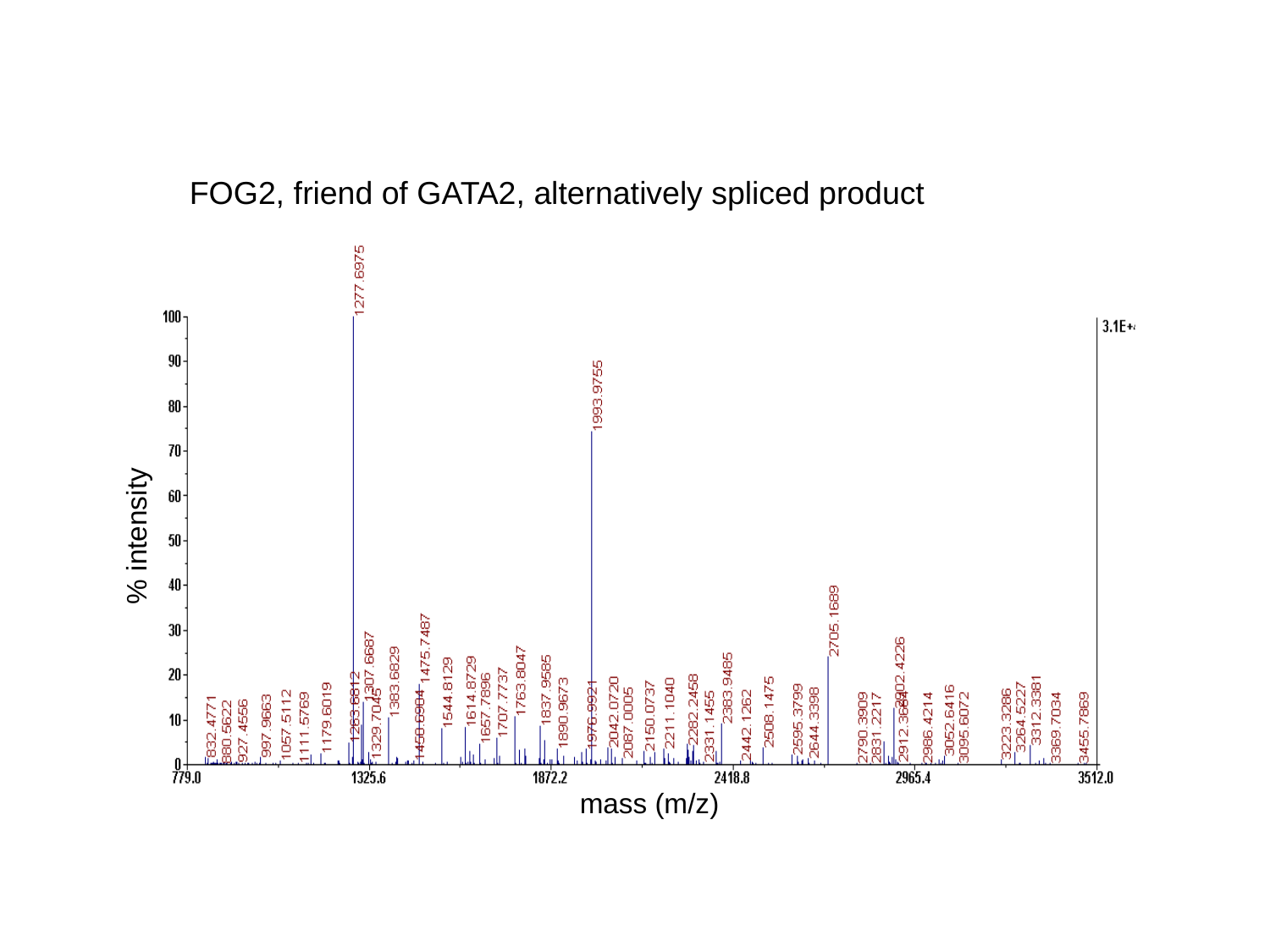

FOG2, friend of GATA2, alternatively spliced product
% intensity
mass (m/z)

## Slide 4
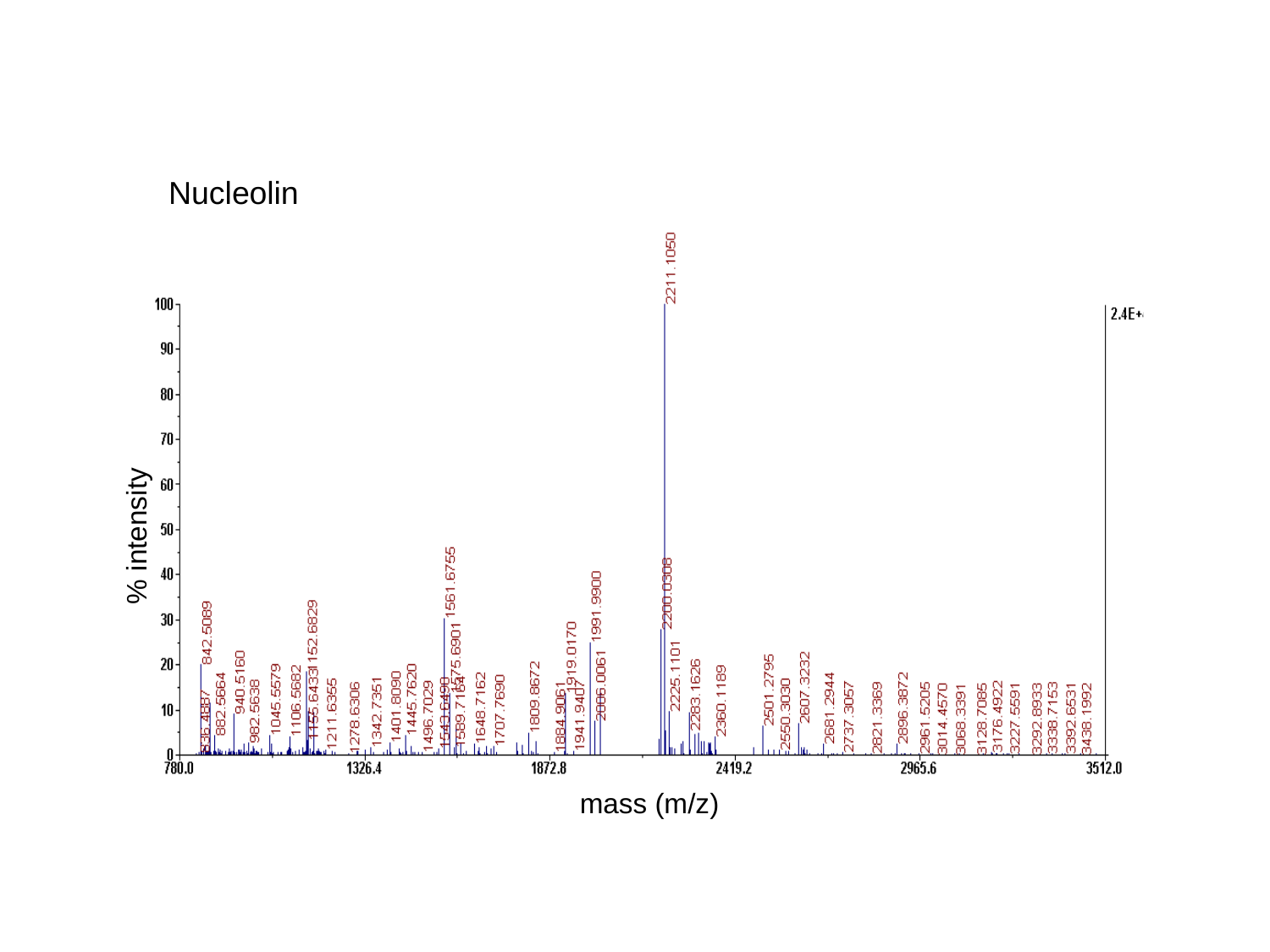

Nucleolin
% intensity
mass (m/z)

## Slide 5
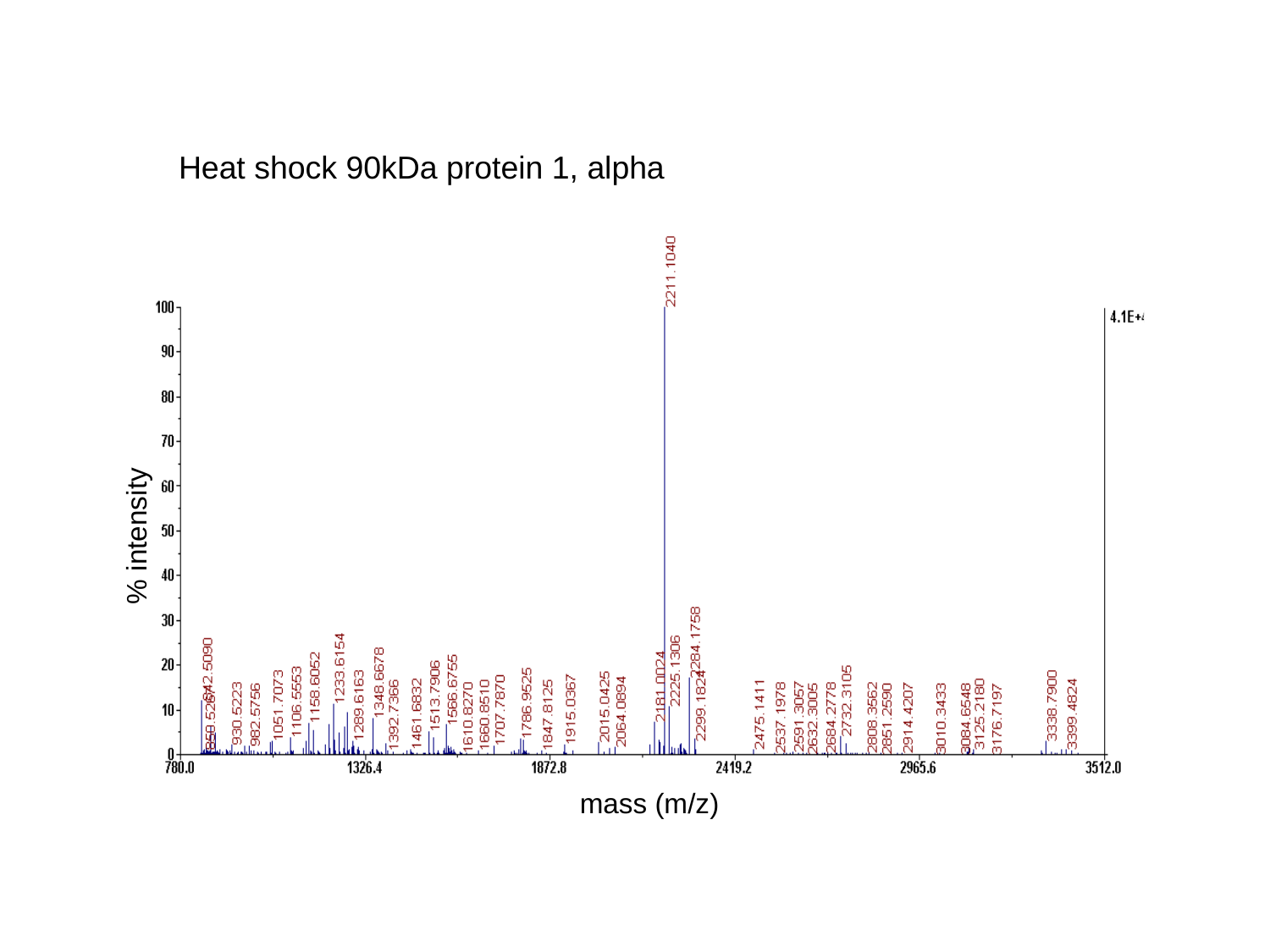

Heat shock 90kDa protein 1, alpha
% intensity
mass (m/z)

## Slide 6
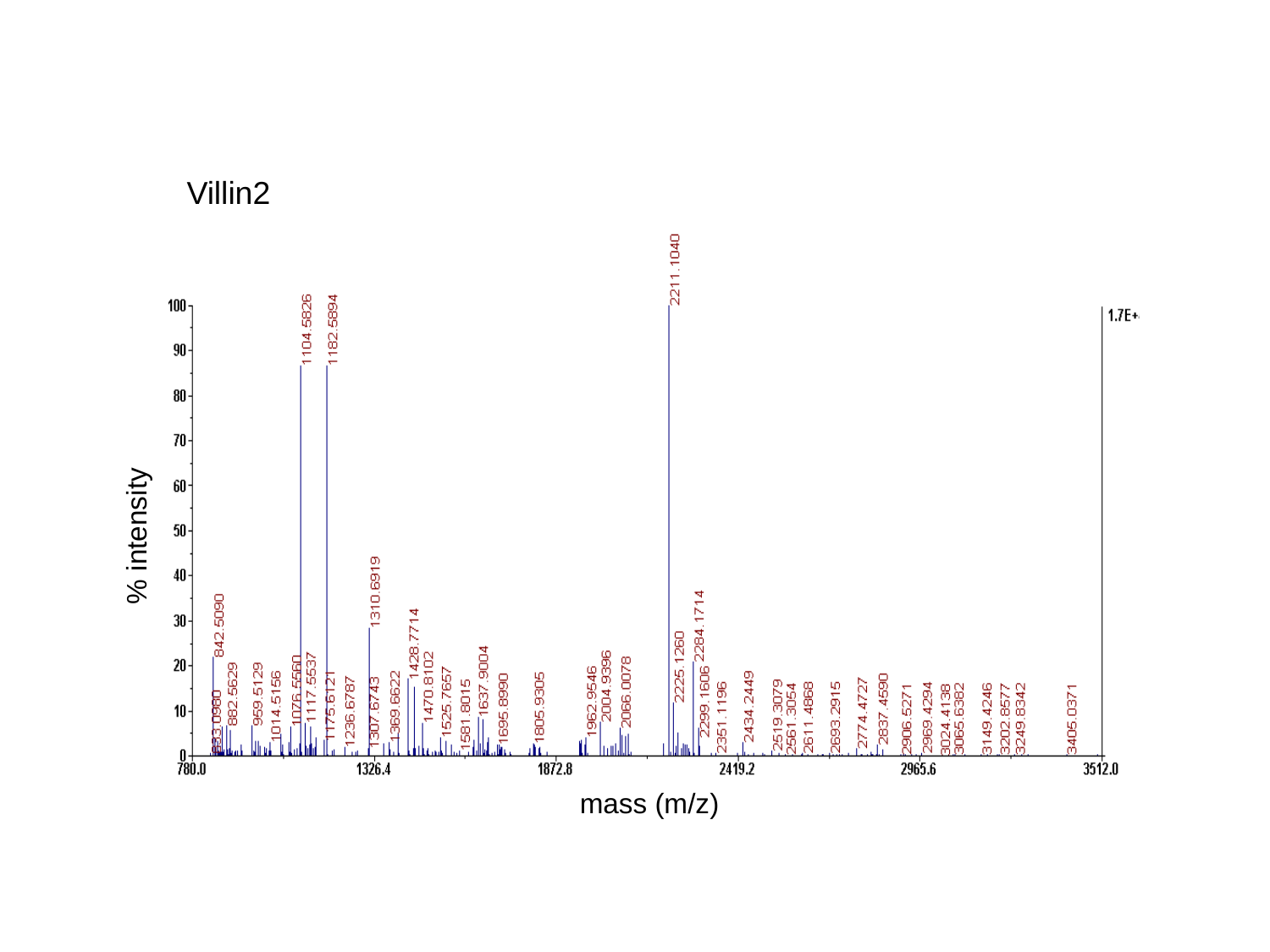

Villin2
% intensity
mass (m/z)

## Slide 7
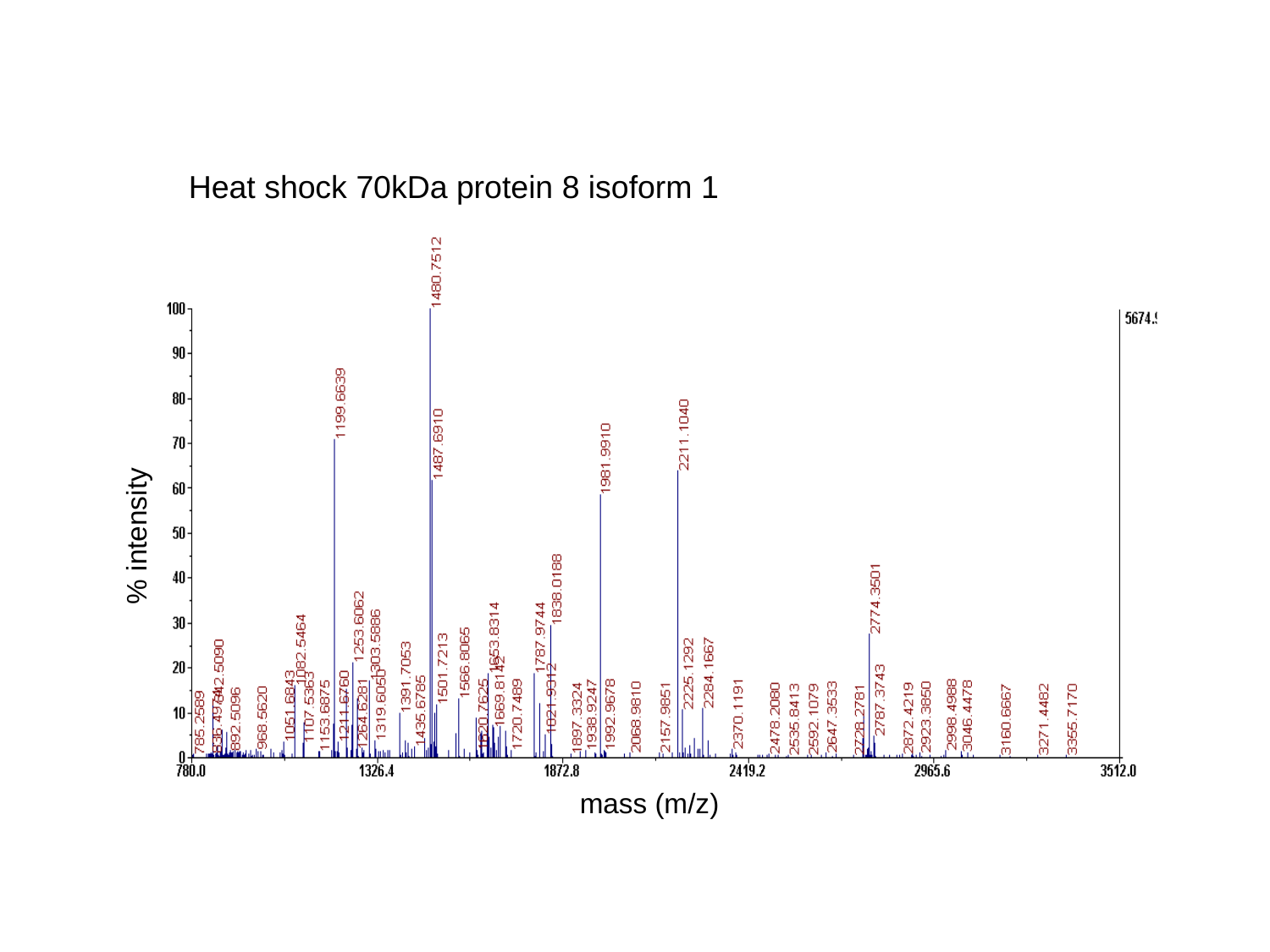

Heat shock 70kDa protein 8 isoform 1
% intensity
mass (m/z)

## Slide 8
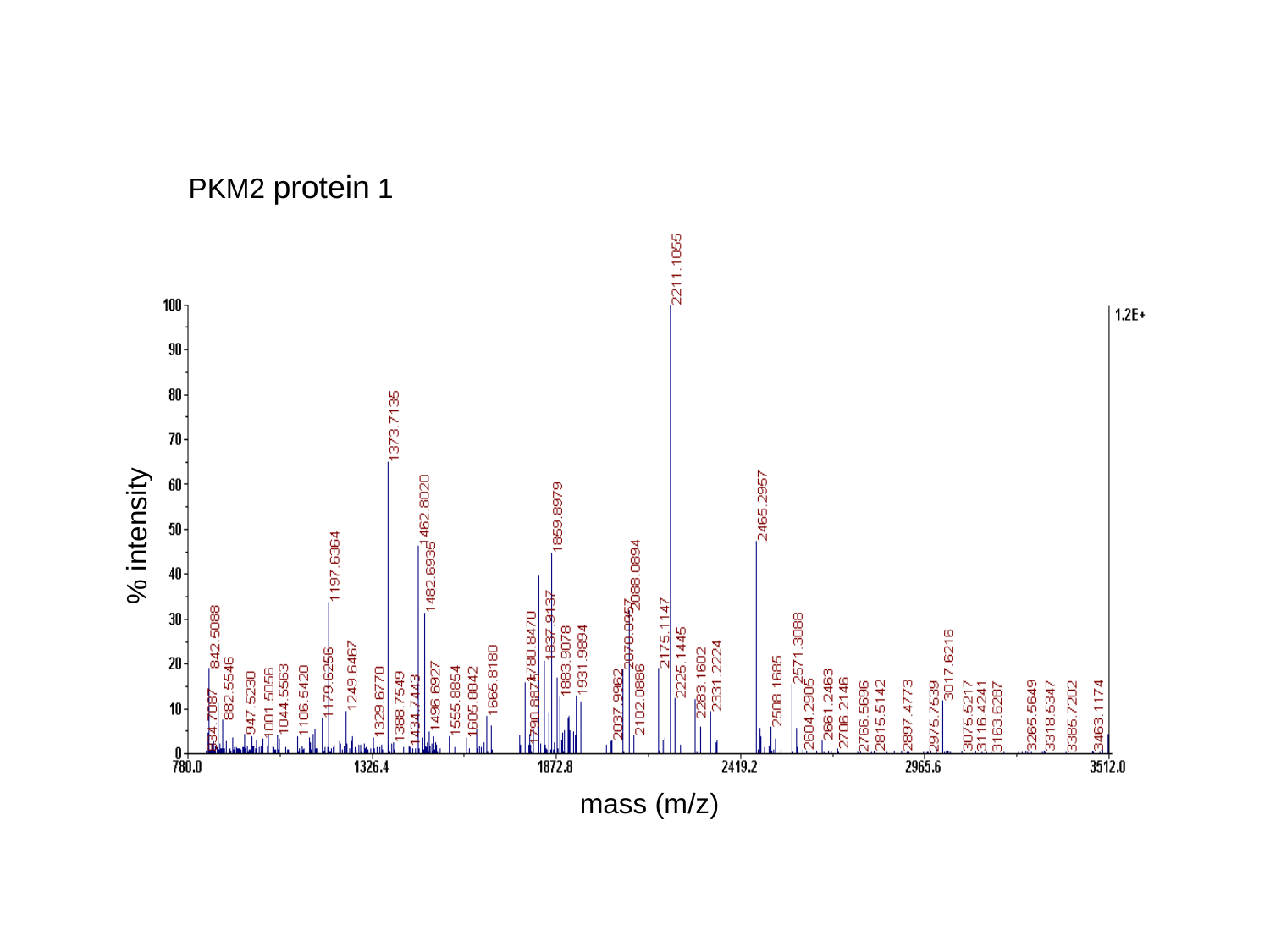

PKM2 protein 1
% intensity
mass (m/z)

## Slide 9
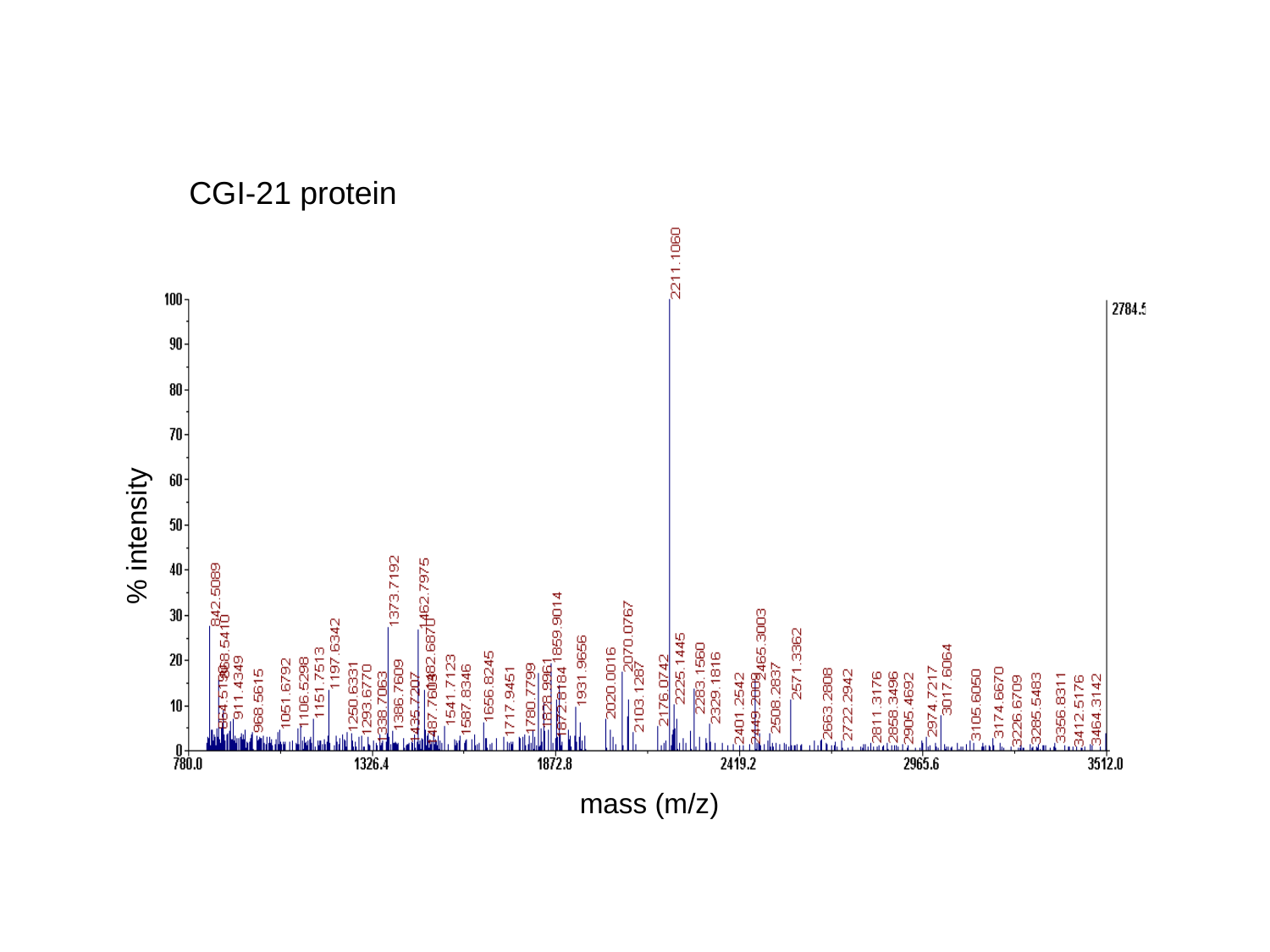

CGI-21 protein
% intensity
mass (m/z)

## Slide 10
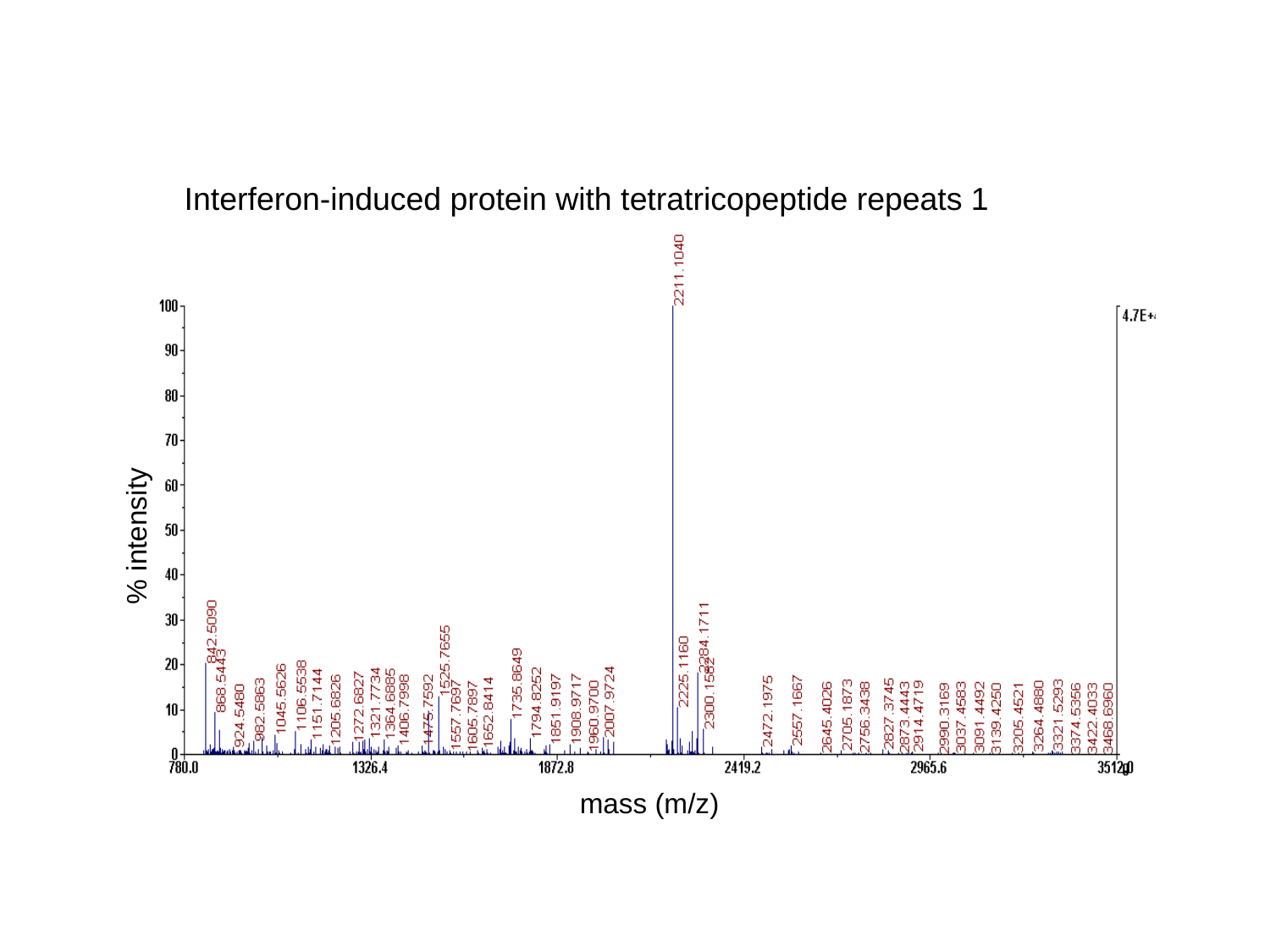

Interferon-induced protein with tetratricopeptide repeats 1
% intensity
mass (m/z)

## Slide 11
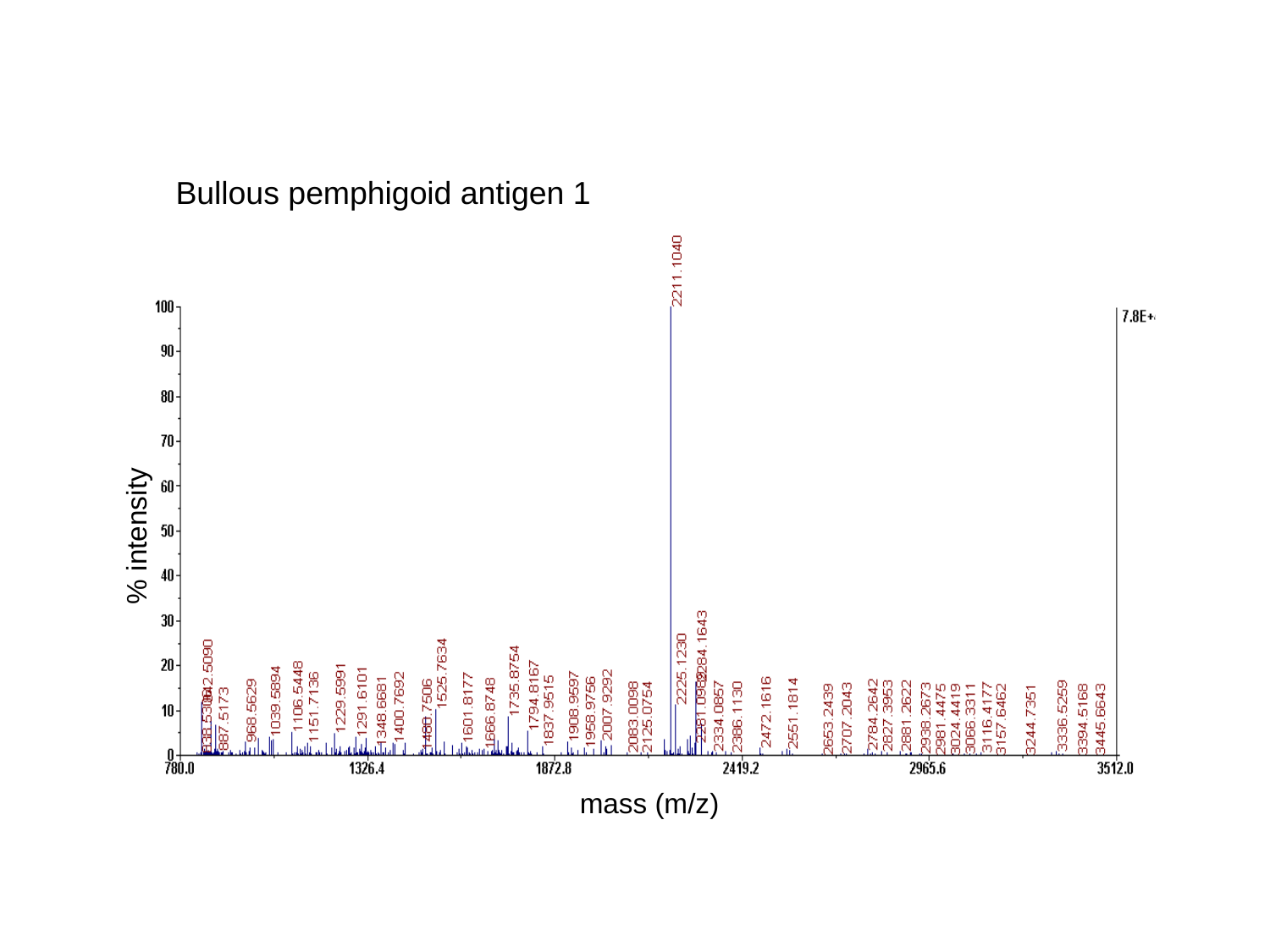

Bullous pemphigoid antigen 1
% intensity
mass (m/z)

## Slide 12
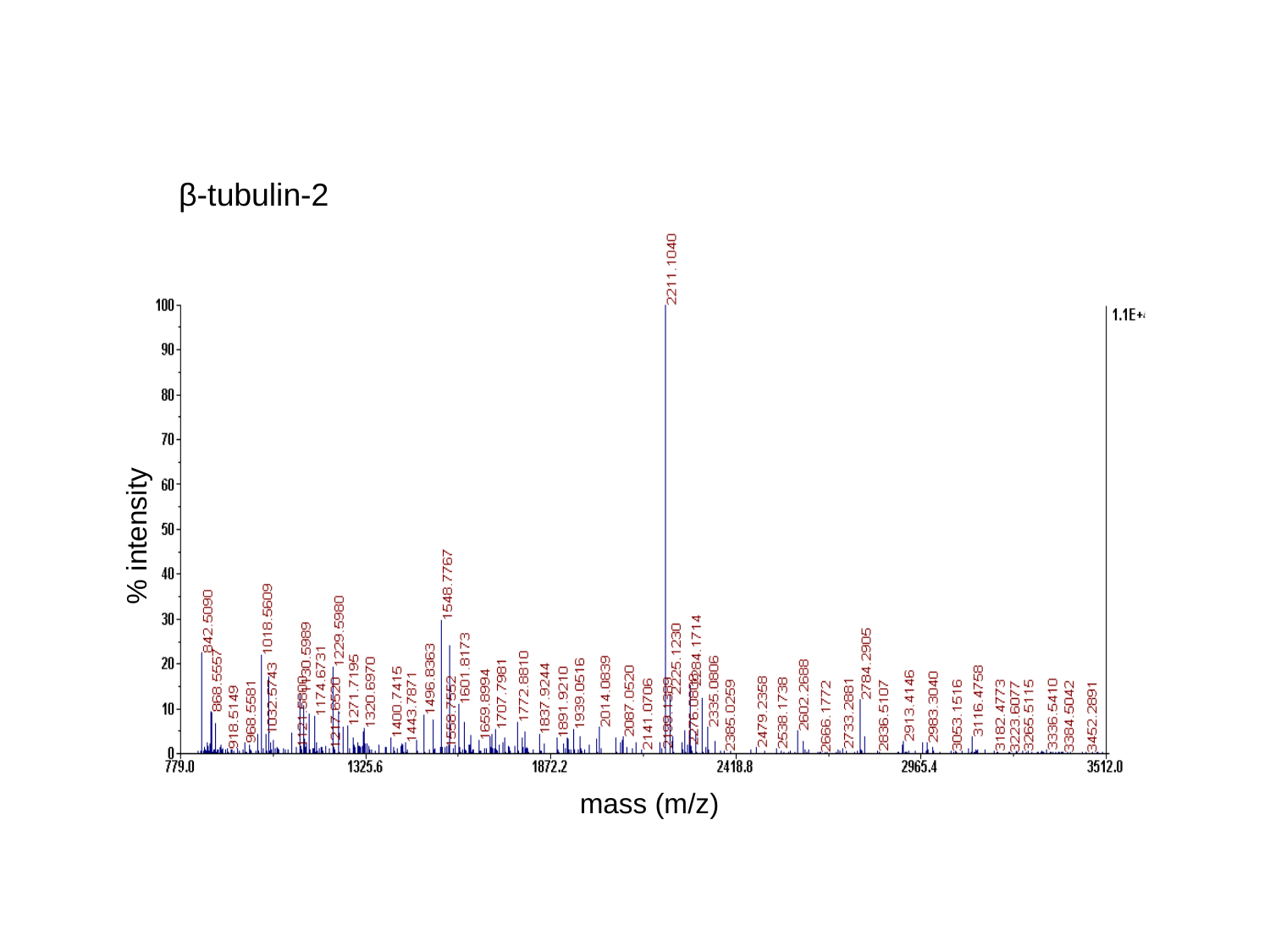

β-tubulin-2
% intensity
mass (m/z)

## Slide 13
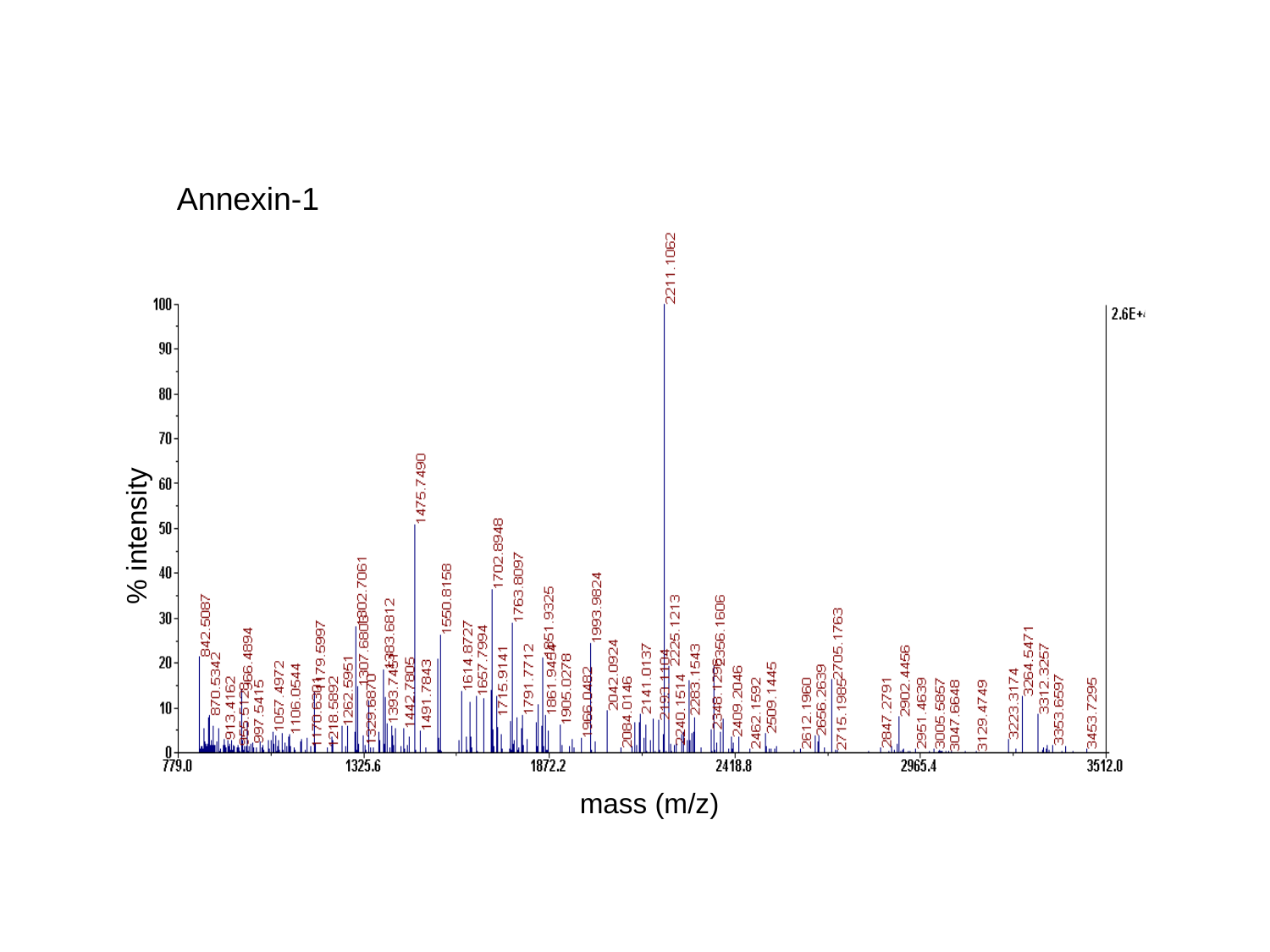

Annexin-1
% intensity
mass (m/z)

## Slide 14
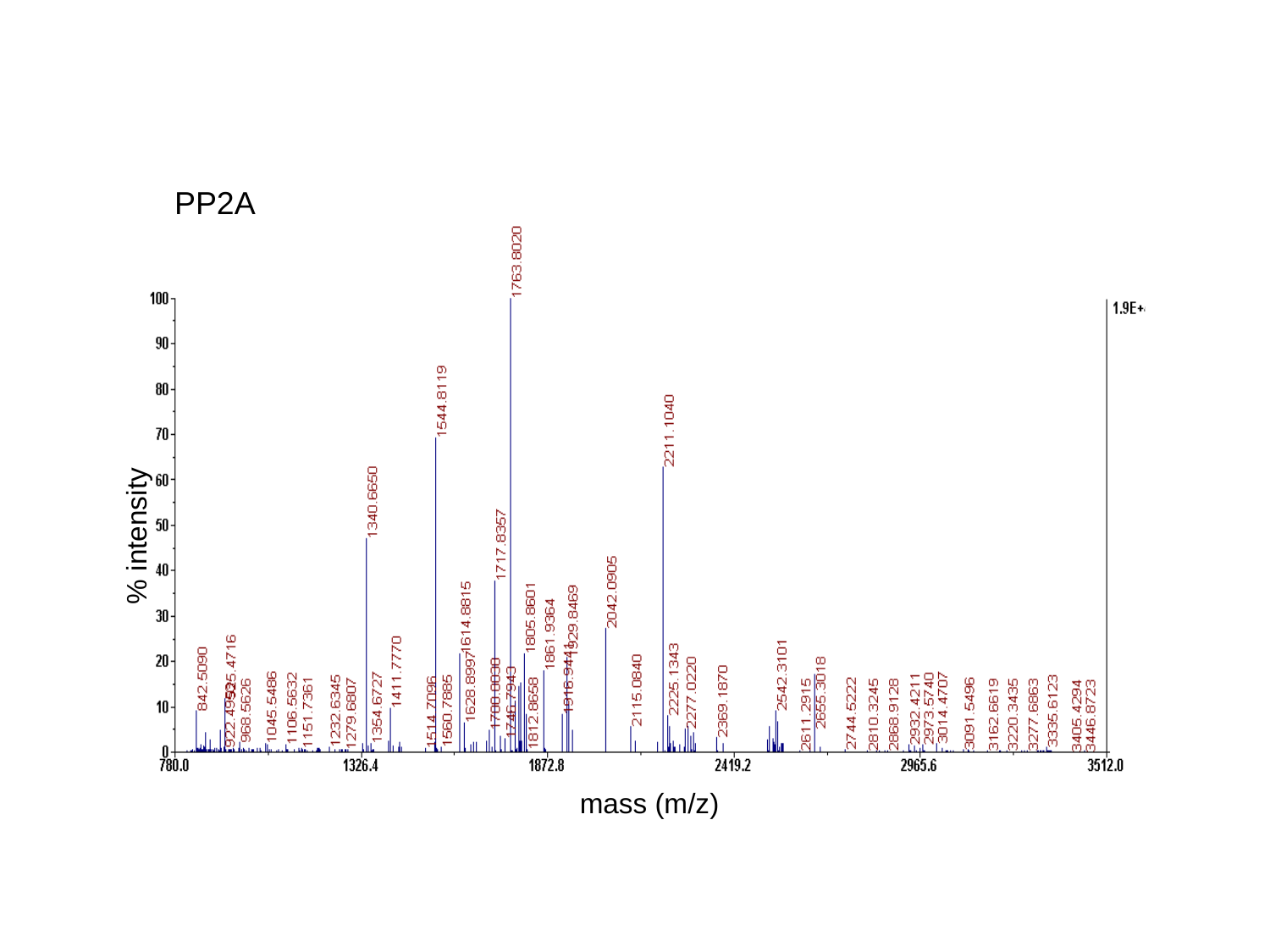

PP2A
% intensity
mass (m/z)

## Slide 15
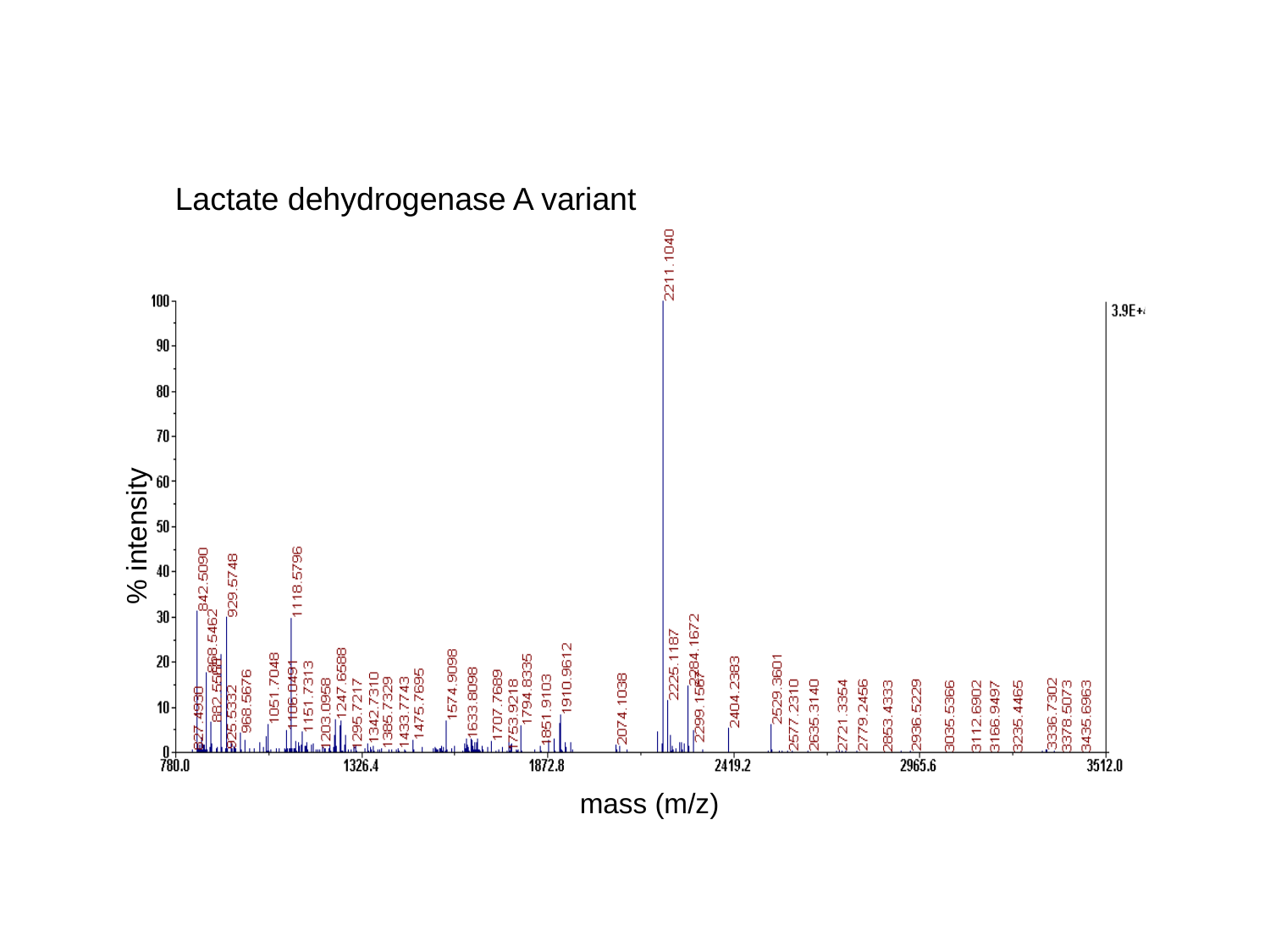

Lactate dehydrogenase A variant
% intensity
mass (m/z)

## Slide 16
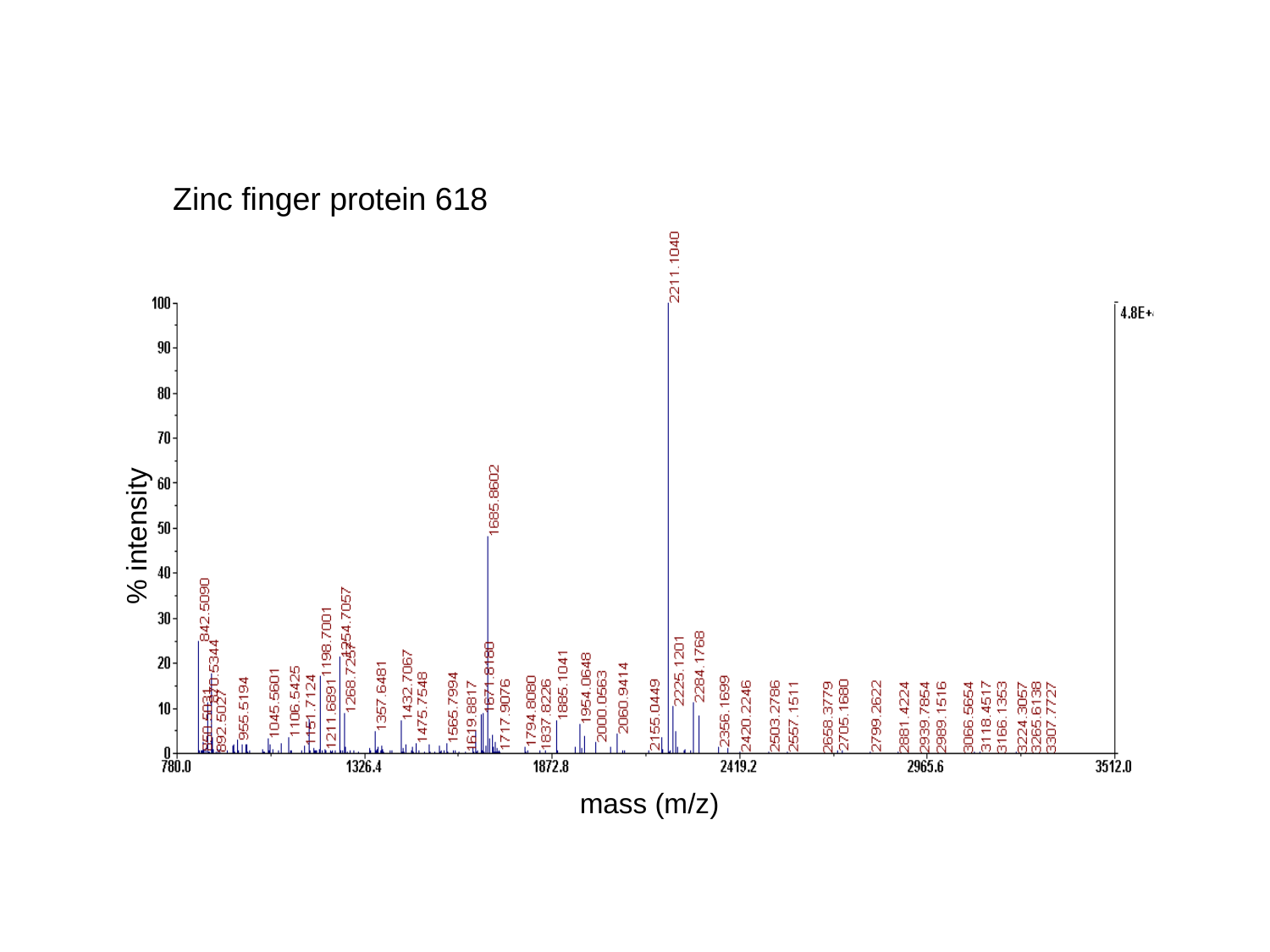

Zinc finger protein 618
% intensity
mass (m/z)

## Slide 17
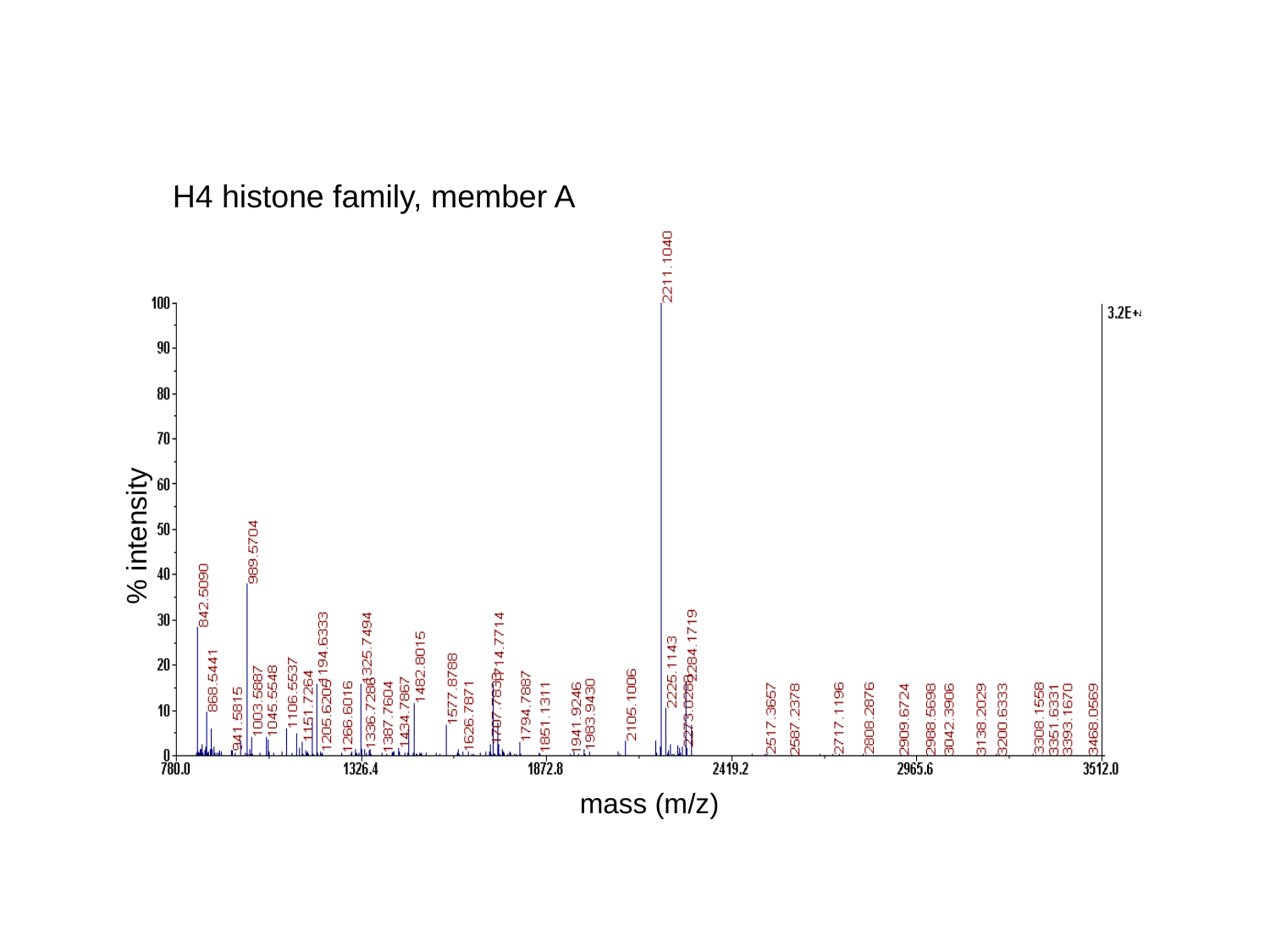

H4 histone family, member A
% intensity
mass (m/z)

## Slide 18
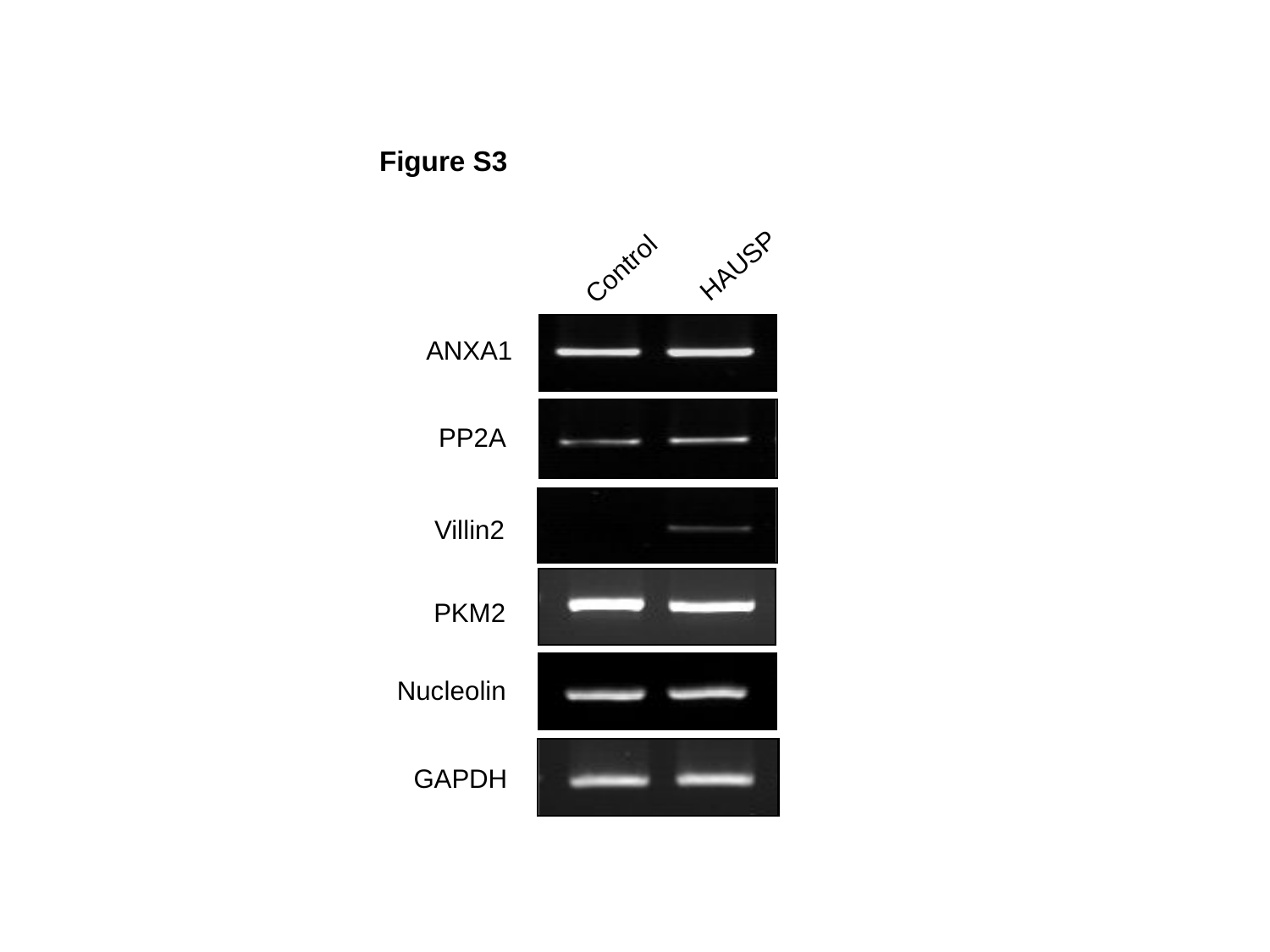

Figure S3
HAUSP
Control
ANXA1
PP2A
Villin2
PKM2
Nucleolin
GAPDH

## Slide 19
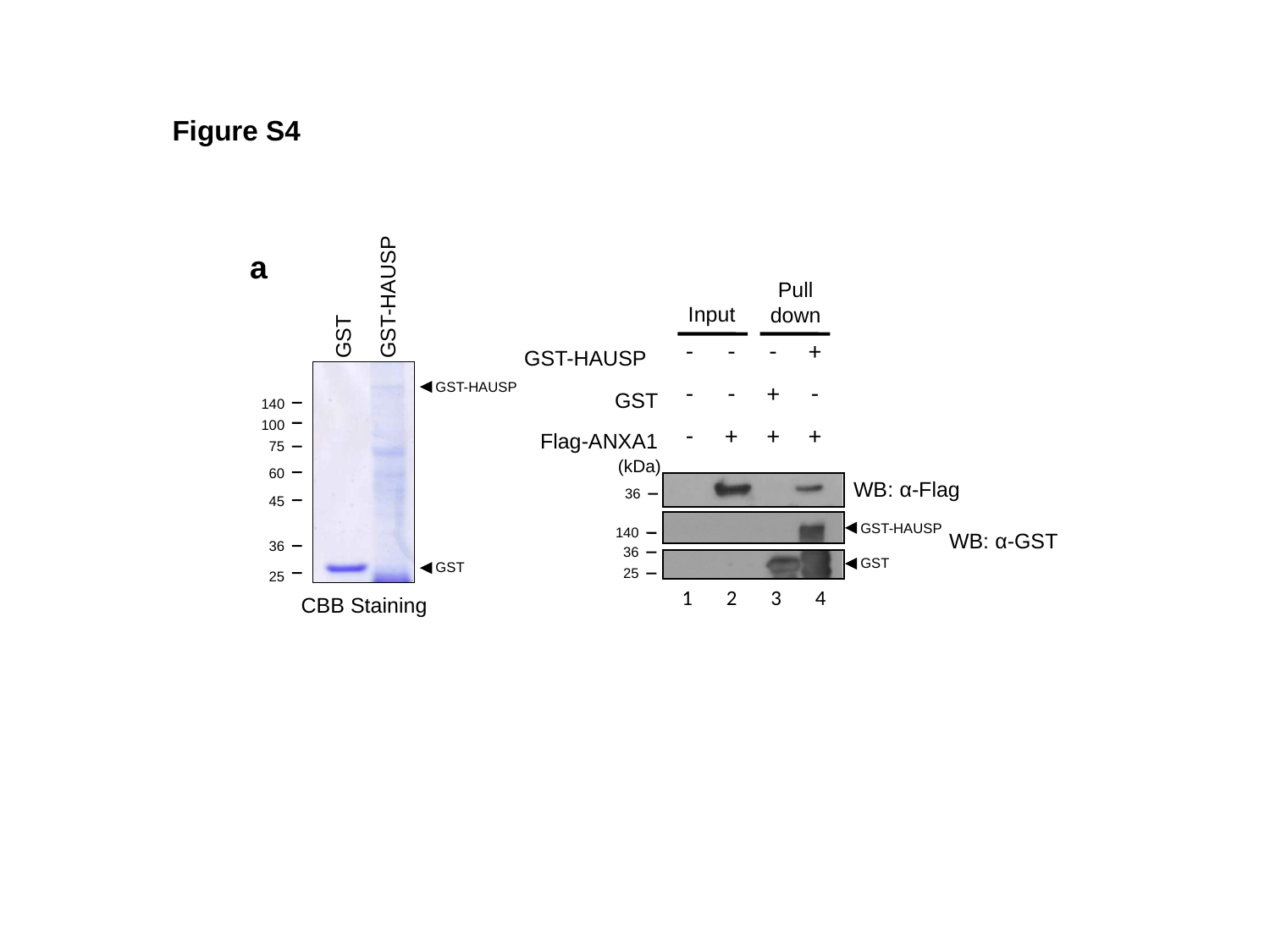

Figure S4
a
Pull
down
GST
GST-HAUSP
Input
| - | - | - | + |
| --- | --- | --- | --- |
| - | - | + | - |
| - | + | + | + |
 GST-HAUSP
GST-HAUSP
GST
140
100
Flag-ANXA1
75
(kDa)
60
WB: α-Flag
36
45
GST-HAUSP
140
WB: α-GST
36
36
GST
GST
25
25
| 1 | 2 | 3 | 4 |
| --- | --- | --- | --- |
CBB Staining

## Slide 20
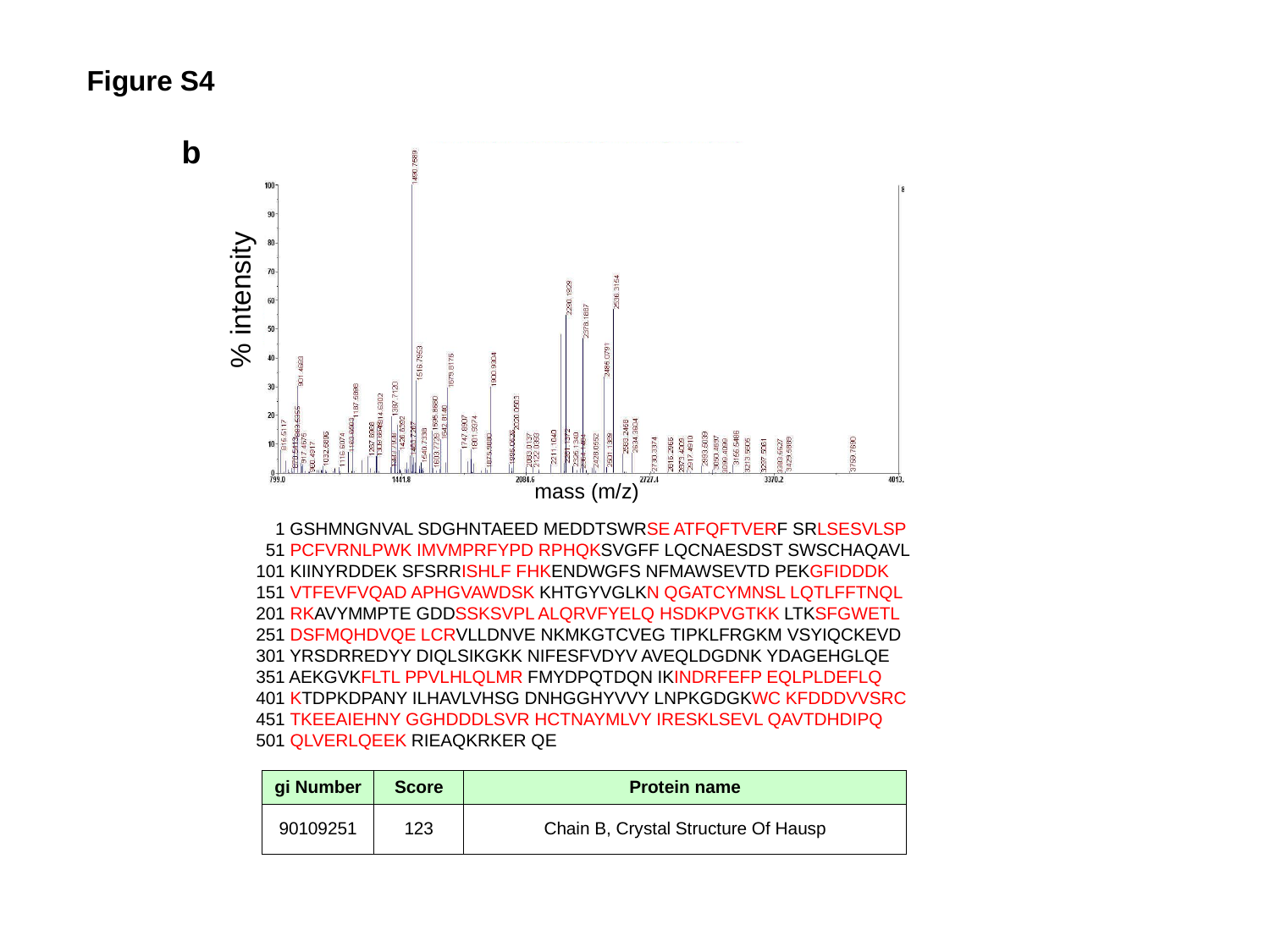

Figure S4
b
% intensity
mass (m/z)
 1 GSHMNGNVAL SDGHNTAEED MEDDTSWRSE ATFQFTVERF SRLSESVLSP
 51 PCFVRNLPWK IMVMPRFYPD RPHQKSVGFF LQCNAESDST SWSCHAQAVL
 101 KIINYRDDEK SFSRRISHLF FHKENDWGFS NFMAWSEVTD PEKGFIDDDK
 151 VTFEVFVQAD APHGVAWDSK KHTGYVGLKN QGATCYMNSL LQTLFFTNQL
 201 RKAVYMMPTE GDDSSKSVPL ALQRVFYELQ HSDKPVGTKK LTKSFGWETL
 251 DSFMQHDVQE LCRVLLDNVE NKMKGTCVEG TIPKLFRGKM VSYIQCKEVD
 301 YRSDRREDYY DIQLSIKGKK NIFESFVDYV AVEQLDGDNK YDAGEHGLQE
 351 AEKGVKFLTL PPVLHLQLMR FMYDPQTDQN IKINDRFEFP EQLPLDEFLQ
 401 KTDPKDPANY ILHAVLVHSG DNHGGHYVVY LNPKGDGKWC KFDDDVVSRC
 451 TKEEAIEHNY GGHDDDLSVR HCTNAYMLVY IRESKLSEVL QAVTDHDIPQ
 501 QLVERLQEEK RIEAQKRKER QE
| gi Number | Score | Protein name |
| --- | --- | --- |
| 90109251 | 123 | Chain B, Crystal Structure Of Hausp |

## Slide 21
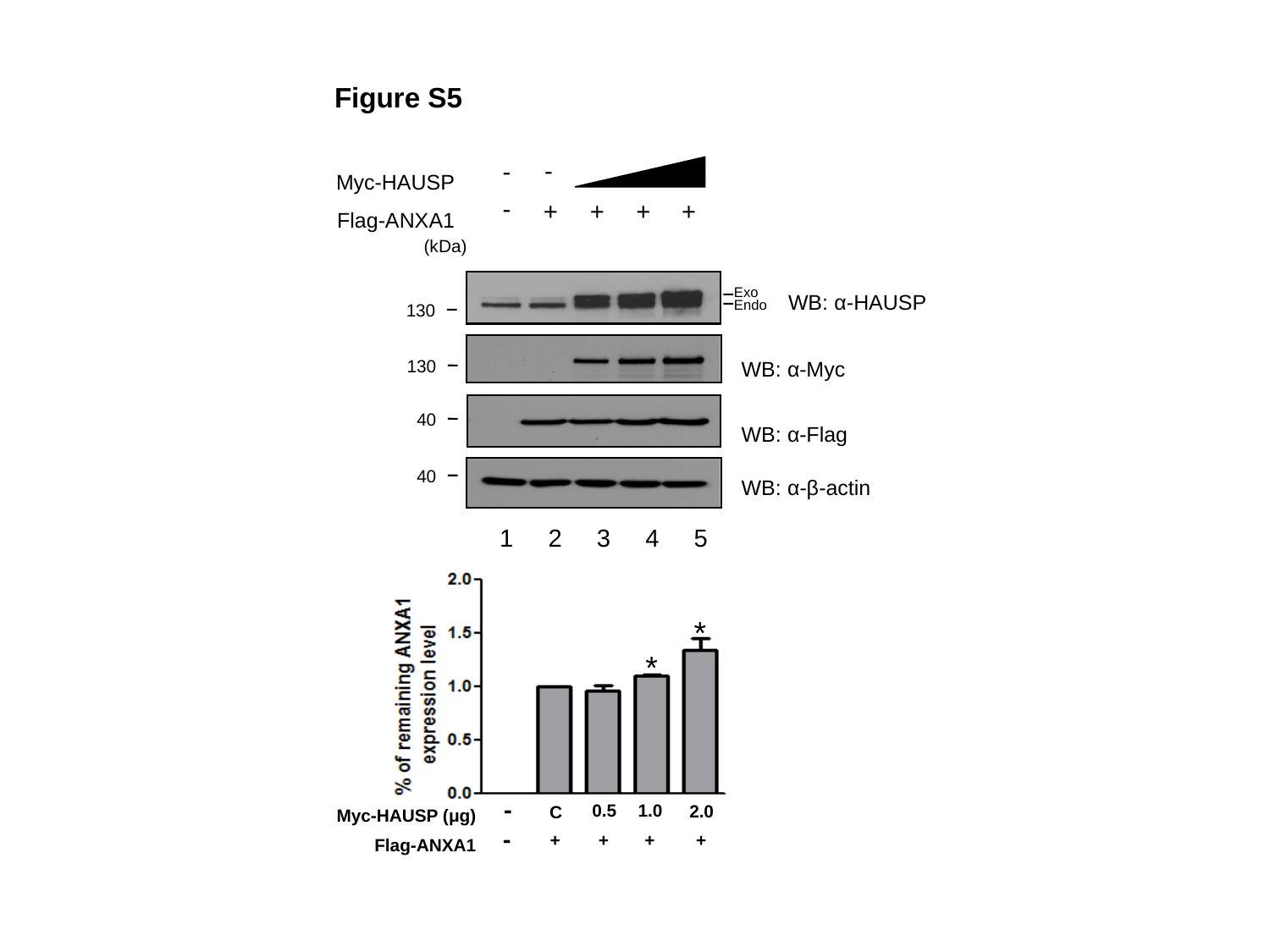

Figure S5
-
-
Myc-HAUSP
Flag-ANXA1
-
+
+
+
+
(kDa)
WB: α-HAUSP
Exo
Endo
130
WB: α-Myc
130
40
WB: α-Flag
40
WB: α-β-actin
1 2 3 4 5
Myc-HAUSP (μg)
-
0.5
1.0
2.0
C
-
Flag-ANXA1
+
+
+
+
*
*

## Slide 22
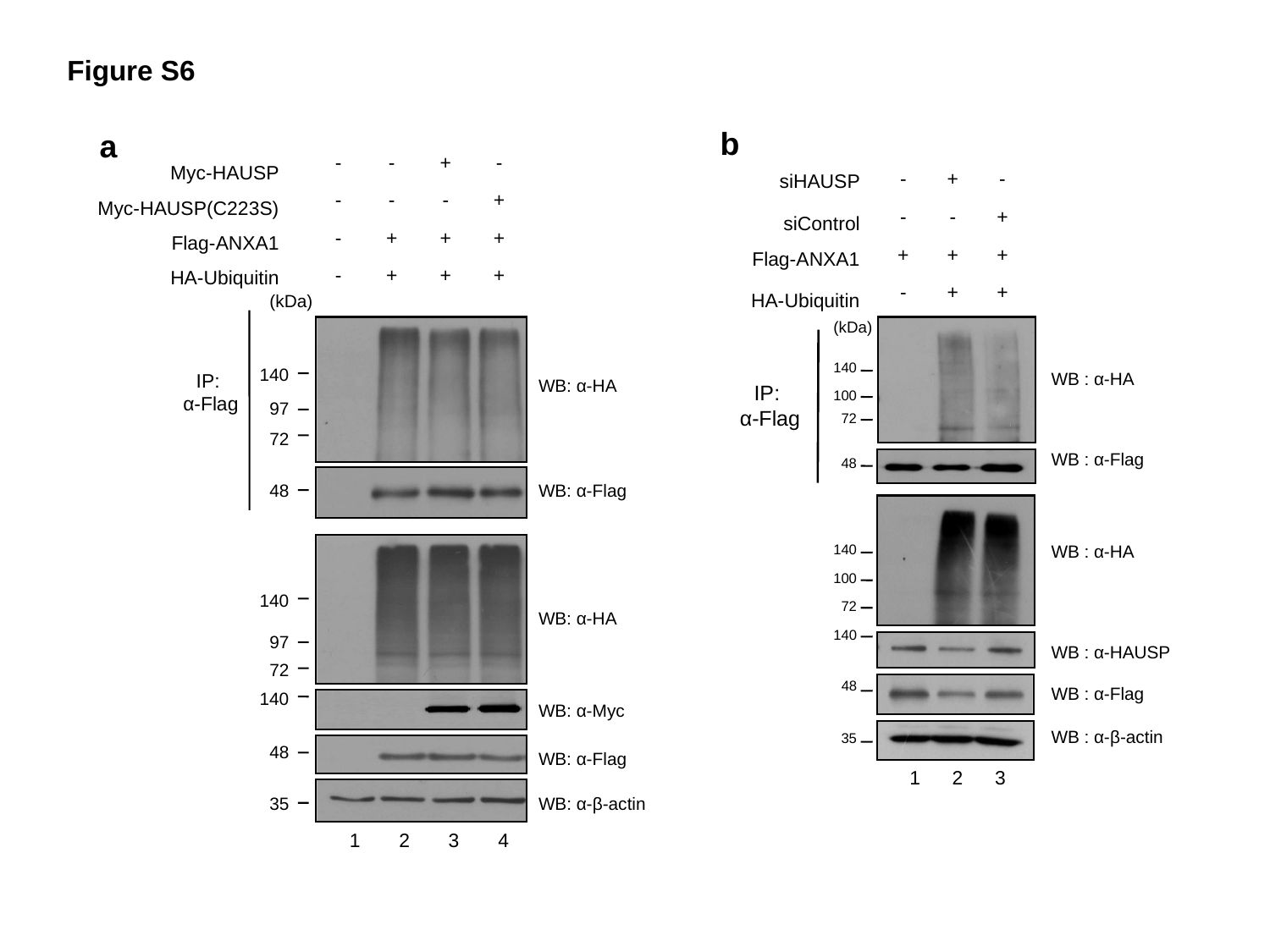

Figure S6
b
a
Myc-HAUSP
Myc-HAUSP(C223S)
Flag-ANXA1
HA-Ubiquitin
| - | - | + | - |
| --- | --- | --- | --- |
| - | - | - | + |
| - | + | + | + |
| - | + | + | + |
siHAUSP
| - | + | - |
| --- | --- | --- |
| - | - | + |
| + | + | + |
| - | + | + |
siControl
Flag-ANXA1
HA-Ubiquitin
(kDa)
(kDa)
140
140
WB: α-HA
WB : α-HA
IP:
α-Flag
IP:
 α-Flag
100
97
72
72
WB : α-Flag
48
WB: α-Flag
48
140
WB : α-HA
100
140
WB: α-HA
72
140
97
WB : α-HAUSP
72
48
WB : α-Flag
140
WB: α-Myc
WB : α-β-actin
35
WB: α-Flag
48
1
2
3
35
WB: α-β-actin
1
2
3
4

## Slide 23
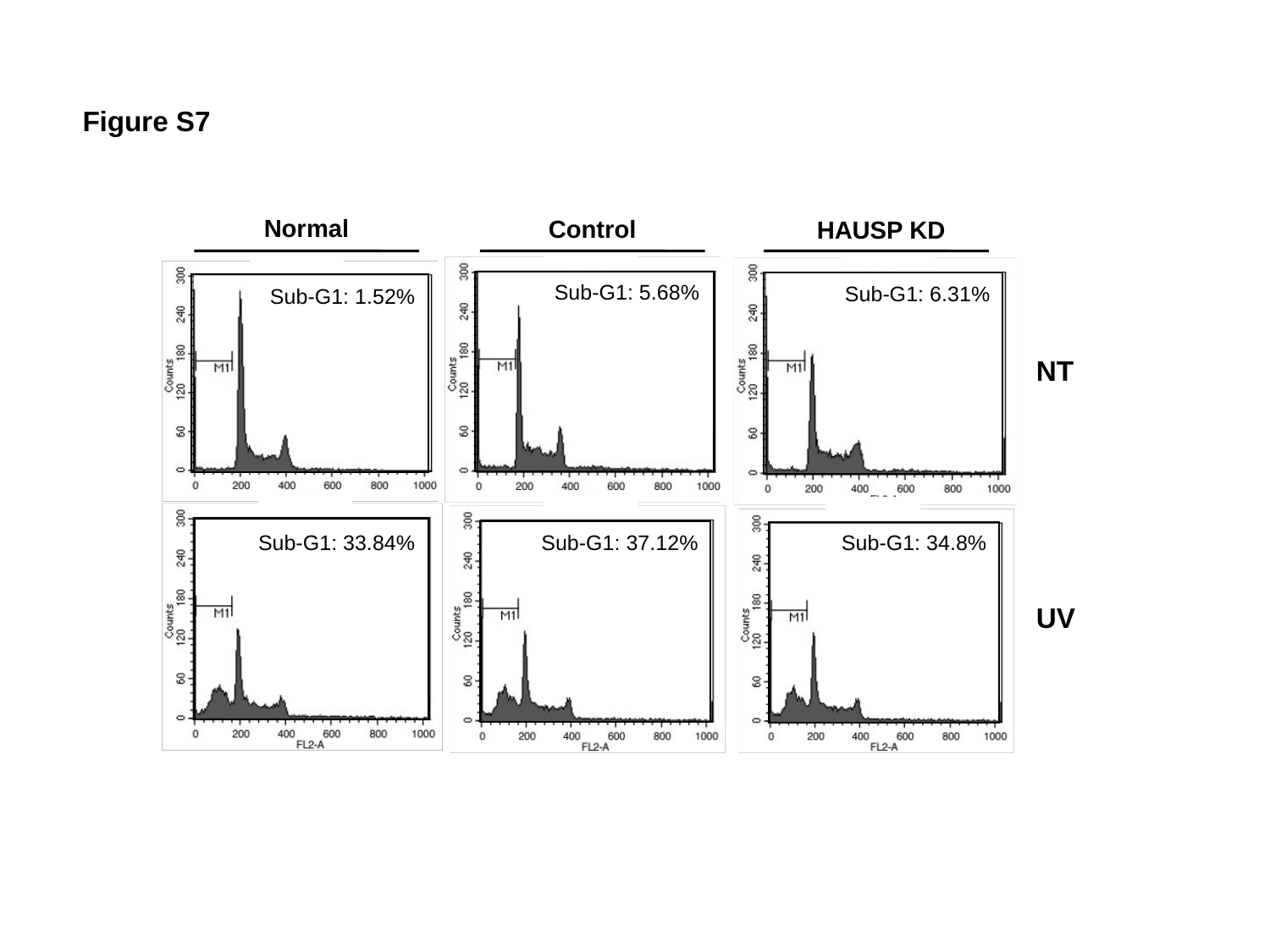

Figure S7
Normal
Control
HAUSP KD
Sub-G1: 5.68%
Sub-G1: 6.31%
Sub-G1: 1.52%
NT
Sub-G1: 33.84%
Sub-G1: 37.12%
Sub-G1: 34.8%
UV
